# Novel role for SLPI in MOG-induced EAE revealed by spinal cord expression analysis

**DOI:** 10.1186/1742-2094-5-20

**Published:** 2008-05-26

**Authors:** Andre M Mueller, Xiomara Pedré, Thomas Stempfl, Ingo Kleiter, Sebastien Couillard-Despres, Ludwig Aigner, Gerhard Giegerich, Andreas Steinbrecher

**Affiliations:** 1 Department of Neurology, University of Regensburg, Franz-Josef-Strauss-Allee 11, 93053 Regensburg, Germany; 2 Centre of Excellence for Fluorescent Bioanalytics (KFB), University of Regensburg, Josef-Engert Str. 11, 93053 Regensburg, Germany

## Abstract

**Background:**

Experimental autoimmune encephalomyelitis (EAE) induced by myelin oligodendrocyte protein (MOG) in female *Dark Agouti *(DA) rats is a chronic demyelinating animal model of multiple sclerosis (MS). To identify new candidate molecules involved in the evolution or repair of EAE-lesions we used Affymetrix oligonucleotide microarrays to compare the spinal cord transcriptome at the peak of EAE, during remission and at the first relapse with healthy DA rats.

**Methods:**

Untreated DA rats and DA rats immunised with MOG protein were sacrificed at defined time points. Total RNA was isolated from spinal cord tissue and used for hybridization of Affymetrix rat genome arrays RG U34 A-C. Selected expression values were confirmed by RealTime PCR.

Adult neural stem cells were incubated with recombinant secretory leukocyte protease inhibitor (SLPI). Proliferation was assessed by BrdU incorporation, cyclin D1 and HES1 expression by RealTime PCR, cell differentiation by immunofluorescence analysis and IkappaBalpha degradation by Western blot.

**Results:**

Among approximately 26,000 transcripts studied more than 1,100 were differentially regulated. Focussing on functional themes, we noticed a sustained downregulation of most of the transcripts of the cholesterol biosynthesis pathway. Furthermore, we found new candidate genes possibly contributing to regenerative processes in the spinal cord. Twelve transcripts were solely upregulated in the recovery phase, including genes not previously associated with repair processes. Expression of SLPI was upregulated more than hundredfold during EAE attack. Using immunohistochemistry, SLPI was identified in macrophages, activated microglia, neuronal cells and astrocytes. Incubation of adult neural stem cells (NSC) with recombinant SLPI resulted in an increase of cell proliferation and of differentiation towards oligodendrocytes. These processes were paralleled by an upregulation of the cell-cycle promotor *cyclin D1 *and a suppression of the cell differentiation regulator HES1. Finally, SLPI prevented the degradation of IkappaBalpha, which may explain the suppression of the cell differentiation inhibitor HES1 suggesting a possible mechanism of oligodendroglial differentiation.

**Conclusion:**

We identified novel features of gene expression in the CNS during EAE, in particular the suppression of genes of cholesterol biosynthesis and a strong upregulation of SLPI, a gene which is for the first time associated with autoimmune inflammation. The capacity of SLPI to increase proliferation of adult NSC and of oligodendroglial differentiation suggests a novel role for SLPI in the promotion of tissue repair, beyond its known functions in the prevention of tissue damages by protease inhibition damage and modulation of inflammatory reactions.

## Background

Experimental autoimmune encephalomyelitis (EAE) represents a variety of animal models reflecting clinical and pathological characteristics of multiple sclerosis (MS). MS is presumably a autoimmune CNS disease with stepwise or chronic progressive evolution of inflammation, demyelination, axonal injury and oligodendrocyte death intertwined with the nervous system's attempts to repair damage and regain homeostasis. The complexity of these processes remains a formidable obstacle to the elucidation of the primary and driving pathogenetic events [1].

Transcriptome studies of CNS tissue in MS and EAE probing many thousand gene products in parallel have resulted in interesting and unexpected target molecules possibly suitable for therapeutic trials in MS [2,3]. Most studies describing the transcriptional spinal cord profile in EAE have been performed in murine models [4-9]. EAE induced by myelin oligodendrocyte protein (MOG) in the rat represents a spectrum of diseases mimicking various forms of MS pathology depending on the selection of strain, gender and experimental procedures, respectively. In female *Dark agouti *(DA) rats, a chronic relapsing EAE variant can be induced, characterized by widespread demyelination, axonal damage and remyelination [10]. MOG has been recognized as a particularly likely candidate for an initial antigen-specific attack in the CNS during MS, and T- and B-cell responses to MOG have been identified in MS-patients and in EAE [11-13]. This report presents for the first time spinal cord transcriptional data of a chronic EAE model in the rat. We compared the mRNA expression profile of more than 26,000 transcripts in spinal cords of DA rats by a large scale gene expression approach using the DNA microarray technique. Expression profiling from spinal cord tissue comparing rats with EAE and healthy control rats was performed in three distinct stages of disease evolution, *i.e*. acute, recovery and relapsing phases of EAE. More than 1,100 significantly regulated transcripts were identified. While confirming well-established features of EAE, we identified several differentially upregulated transcripts not described previously. In more detail, we examined the secretory leukocyte protease inhibitor (SLPI) representing the most strongly upregulated gene in this study. SLPI is a homeostatic protein known to be expressed at mucosal surfaces by epithelial cells, macrophages and neutrophils. It is involved in the resolution of inflammation by suppressing protease activity, by attenuating innate immune responses and by inhibiting the activation and proliferation of B cells [14,15]. In the CNS its induction has been reported as a consequence of ischemic stroke [14] and spinal cord injury [16]. In this study we provide evidence that SLPI promotes oligodendroglial proliferation and differentiation. We suggest that SLPI may have a novel and multiple roles in CNS inflammation, i.e. inhibition of pathogenic proteases, immunomodulation and promotion of CNS repair.

## Material and methods

### Animals

Female dark agouti (DA) rats, 6–8-week old, were purchased from Harlan Winkelmann. They were housed in the animal facility of the University of Regensburg and were 8–10-weeks old when used for the experiments. All procedures were conducted according to protocols approved by the animal care committee of the Medical Faculty.

### Induction and clinical evaluation of EAE

For the microarray experiment 20 female DA rats were immunized intradermally at the base of the tail with 65 μg MOG(aa 1–125) emulsified in complete Freund's adjuvant (CFA) containing 400 μg of heat-inactivated *Myc. tuberculosis *(H37Ra, (DIFCO)) in a total volume of 200 μl.

Animals were weighed and scored daily for signs of EAE according to the following scale: 0, no disease; 1, tail paralysis; 2, hind limb weakness; 3, hind limb paralysis; 4, hind limb paralysis plus forelimb weakness; 5, moribund or dead. The mean cumulative score for a treatment group was calculated as the sum of the daily scores of all animals from day zero until the end of the experiment divided by the number of animals in the respective group.

### Microarrays

The Affymetrix *GeneChip Rat Genome U34 *Arrays A, B and C were used for this study. They represent more than 26,000 genes currently consisting of approximately 8,000 characterised genes and 16,000 established sequence tags (ESTs).

### Sample preparation and hybridization

DA rats were perfused with PBS to remove circulating blood cells and their spinal cords and inguinal lymph nodes were collected. Total RNA was extracted from lumbar spinal cords and lymph nodes, respectively, using the RNeasy Lipid Tissue Midi Kit (Qiagen) and examined for signs of degradation with the Bioanalyzer 2100 (Agilent Technologies). All further preparation steps were performed at the "Centre of Excellence for Fluorescent Bioanalytics" (KFB, Regensburg, Germany). Isolated RNA was processed according to the standard Affymetrix protocol. Hybridization, washing and staining of Affymetrix rat U34 arrays were carried out following manufacturer's instructions. In brief, double stranded cDNA was generated from 10 μg RNA with the Superscript Double-Stranded cDNA Synthesis Kit (Invitrogen). The High Yield RNA Transcript Labeling Kit (Enzo Life Sciences) was used to obtain biotinylated cRNA. The three Affymetrix arrays RG U34A-C were consecutively hybridised with the samples in a rotating chamber (16 h, 45°C). The arrays were scanned by the G2500A GeneArray Scanner (Affymetrix).

### Data analysis

Hybridization patterns were processed and quantified using MAS 5.0 software. Parameters for the assessment of the hybridization quality included the signal-to-noise ratio, signal spreading, average signal intensity and the ratio between the 5' and 3'ends of the house keeping genes actin and GAPDH. Empirical cut-off values were set for these criteria and samples not meeting them were excluded from further analysis.

Transcripts labelled *absent *by the dchip 2006 algorithm [17] in more than 80% of the samples were also not subjected to further analysis. Animals from the different disease phases were compared with healthy control animals using dchip 2006. Significantly regulated transcripts had to meet the following criteria: (a) a mean signal ratio > 2 between groups of arrays [6,18-20] and (b) a p-value < 0.01 according to an unpaired t-test.

A functional analysis combined with an extraction of overrepresented functional gene groups was performed using the NetAffx-software (Affymetrix, [21]) and the EASE program [22]. Principal component analyses based on the hybridization results from the RG U34A chips were performed using BRB ArrayTools developed by Dr. Richard Simon and Amy Peng Lam (NIH, USA, [23]).

### Data accession

All data are available online through Array Express (accession number E-MEXP-1025).

### Determination of transcripts expressed in the inflamed CNS but not by non-activated lymph node cells

Transcripts found in the spinal cord of EAE animals are expressed in either CNS resident cells or infiltrating leukocytes. As an approximation to focus on the subset of transcripts derived from CNS resident cells and infiltrating cells "responding" to EAE, genes expressed in lymph node cells (LNC) were *in silico *subtracted from the list of genes expressed in the spinal cords of EAE animals. "Lymph node transcripts" were compiled by hybridization of microarrays with RNA from inguinal lymph nodes of five untreated, healthy DA rats as described above.

### RealTime-PCRs

The regulation of selected genes was validated by quantitative RealTime-PCR using RNA isolated from rats from a different experiment as those sacrificed for microarray hybridization. RNA samples were reverse transcribed with random hexamer primers using the Reverse Transcription System (Promega). RealTime-PCRs were performed with the Mx3005P Cycler (Stratagene) and the QuantiTect SYBR GreenPCR-Kit (Qiagen). RealTime-PCRs were done in triplicate preparations. RealTime-PCR primers are listed in Table 1. Missing primers were purchased from Qiagen (QuantiTect-primer assays, Gene Globe database, Qiagen). The quantity of each transcript was correlated to the amount of 18S rRNA determined by Taqman probes (Applied Biosystems).

**Table 1:** Primer sequences used for RealTime-PCR

Gene	Sense	Antisense
SLPI	TCC CAT TCG TGG ACC AGT GAA GAA	TGC CAT CAC ACT GGC CGT CAT TCT
MOG	TAC AAC TGG CTG CAC CGA AGA CT	AGG CTT TCC TTC GCT CCA GGA AGA
DORA	GAG ATA TGT GGA AAC AAG TCA TCA GC	AAG TCA GCA CAT CTA TGG TCT TCC A
GPNMB	AGG ATT CCA TCT ACA ATT GTG ATG GT	CCT AGT CCC TCT TTA ATG CCT ACT
BAFF	TCC TGC TAC TCC GCT GGC AT	GTC GTC TCC GTT CCG TGA AA
CXCL13	TTT TCT GGA CCA AGG CCA AGA	GGA GCT TGG GGA GTT GAA GTA
IRF-1	CAA GGA GGA ACC AGA GAT CGA CA	TAG AGT TGC CCA GCA GGG TGT C
Lipocalin2	CAA GTG GCC GAC ACT GAC TA	GGT GGG AAC AGA GAA AAC GA
HES1	TAC CCC AGC CAG TGT CAA CA	TTC ATT TAT TCT TGC CCG GC

### Determination of cholesterol concentration in spinal cord tissue

Proteins and lipids were extracted from spinal cord tissue by homogenisation of frozen tissues and sonification in RIPA buffer (150 mM NaCl, 1% (w/v) Igipal, 0.5% (w/v), Na-deoxycholate, 0.1% (w/v), SDS, 50 mM Tris-HCl, pH 7.5).Protein concentrations were determined (Lowry-method) and adjusted to 1 mg/ml using RIPA-buffer. Total cholesterol content was determined by a colorimetric assay (Roche).  

### Immunohistochemical analysis of SLPI expression

Rats were transcardially perfused with ice-cold saline followed by 4% paraformaldehyde (PFA). Spinal cords were removed and postfixed in PFA overnight at 4°C. Tissues were then cryoprotected by 24 h immersion in a 30% (w/v) sucrose/PBS-solution and cut into 30 μm sections using a cryostat. Sections were stored at -80°C in cryoprotectant solution (ethylene glycol, glycerol, 0.1 M phosphate buffer pH 7.4, 1:1:2 by volume).

Double-labelling immunofluorescence was carried out using anti-SLPI (1:200, rabbit anti-human SLPI, HyCult Biotechnology) in combination with either mouse anti-rat monocytes/macrophages (ED1, 1:250; Chemicon), sheep anti-von Willebrand Factor (Serotec, 1:1000), mouse anti-glial fibrillary acidic protein (GFAP, 1:1000; Chemicon) or mouse anti-neuron specific nuclear protein (NeuN, 1:1000; Chemicon). Confocal microscopy was used to determine the cellular localisation of SLPI in spinal cord slices.

### Cultivation of adult neural stem cells (NSC)

Neural stem cells were obtained from the subventricular zone (SVZ) of 4- or 6-week-old male Wistar rats. Animals were sacrificed, and brains dissected and washed in ice-cold Dulbecco's PBS (DPBS) containing 4.5 g/L glucose (DPBS/Glc). The SVZ from six animals were dissected, washed in 10 ml DPBS/Glc and centrifuged for five minutes at 1,600 g at 4°C. Tissue was minced using scissors. Pieces were washed again and centrifuged at 800 g and the pellet resuspended in 0.01% (w/v) papain, 0.1% (w/v) Dispase II (Roche Diagnostics), 0.01% (w/v) DNase I and 12.4 mM manganese sulphate in HBSS (PAA Laboratories). Subsequently the tissue was incubated for 40 min at RT. Thereafter, the suspension was centrifuged at 4°C for 5 min at 800 g and the pellet washed three times in 10 ml DMEM/Ham's F-12 medium containing 2 mM L-glutamine and 100 U/ml penicillin/streptomycin. Cells were then resuspended in 1 ml growth medium (neurobasal medium containing B27 (Invitrogen), 2 mM L-glutamine, 100 U/ml penicillin/streptomycin, 20 ng/ml EGF, 20 ng/ml bFGF and 2 μg/ml heparin). Cells were seeded in 6-well plates coated with 250 μg/ml poly-L-ornithine and 5 μg/ml laminin (Sigma Aldrich) at a density of 25,000 to 100,000 cells/ml and incubated at 37°C in 5% CO_2_. Two thirds of the medium volume were changed weekly [24]. The determination of the purity and of the differentiation potential of the progenitor cells was described before [25]

### Analysis of neural stem cell proliferation

The proliferation of NSC was assessed with the FITC BrdU Flow Kit (BD Pharmingen) according to the manufacturer's instructions. 10,000 cells/well were seeded into laminin-coated 24-well plates in growth medium for four days. After one day the specified amounts of recombinant SLPI (provided by Amgen, USA) [26] or recombinant VEGF (R&D Systems) were added. After three days the cells were pulsed with BrdU (10 μM) for eight hours. The proportion of the BrdU-positive cells was determined using the FACSCalibur-Cytometer (BD Biosciences).

### Western blot analysis of IκBα degradation

300.000 NSC were incubated in 2 ml neurobasal medium with 10 ng/ml TNFα (Peprotech) with or without 500 ng/ml SLPI for the incubated time period. Total protein was extracted using the M-Per-reagent (Pierce Biotechnology), separated by SDS-PAGE and blotted onto a nitrocellulose membrane (Schleicher & Schuell). A mouse anti-IκBα antibody (Cell Signaling Technology) was used to detect IκBα and a mouse anti-actin monoclonal antibody (clone G4.18, Research Diagnostics) for standardisation. The secondary anti-mouse antibody (Chemicon, USA) was conjugated to horseradish peroxidase. Bands were detected using the Immobilon Western substrate (Millipore).

### Assessment of cyclin D1 and HES1 expression in NSC and confirmation of the specificity of the effects of SLPI on NSC

100,000 NSC/well were seeded onto laminin-coated 6-well plates in 2 ml growth medium. After one day the specified amounts of recombinant SLPI, recombinant VEGF (R&D Systems) or recombinant α_1_-antitrypsin (α_1_-AT, Sigma Aldrich) were added. Three days later the cells were harvested, RNA was isolated, cDNA generated and RealTime-PCRs for cyclin D1 were performed with a QuantiTect-primer assay (Gene Globe, Qiagen). The expression of HES1 was determined accordingly in SLPI treated NSC cultures, but the cells were incubated for additional four days in differentiation medium.

For the confirmation of the specificity of SLPI's effects on NSC cultures, NSCs were incubated with 200 ng/ml SLPI for three days as described above with or without 2 μg/ml rabbit anti-human SLPI-antibodies (HyCult Biotechnology). For the evaluation of the specificity of SLPI's effects, the expression of cyclin D1 by NSCs was determined by RealTime-PCR.

### Analysis of cell differentiation and cell death

Fixed NSCs were cultivated for three days with the specified amounts of SLPI in growth medium and subsequently for seven days in differentiation medium (NB/B27 medium with 5% FCS). Afterwards, the cells were washed in TBS buffer and blocked with a TBS-solution containing 1% bovine serum albumin (BSA) and 0.2% Teleostean gelatin (fish gelatin buffer (FGB), Sigma). The same solution was used during the antibody staining. Fluorochrome-conjugated secondary antibodies were used for immunodetection. The following antibodies and final dilutions were used: primary antibodies: rabbit anti-GFAP 1:1000 (DAKO), rabbit anti-GalC 1:200 (Chemicon), mouse anti-βIII-tubulin 1:500 (clone 5G8, Promega); secondary antibodies: donkey anti-mouse or anti-rabbit conjugated with Alexa Fluor^® ^488 (Molecular Probes). Nuclear counterstaining was performed with 4', 6'-diamidino-2-phenylindole dihydrochloride hydrate (DAPI, Sigma) at 0.25 μg/ml. Specimens were mounted on microscope slides. To assess cell death, 50 μg/ml propidium iodide (PI, Sigma) was added to the culture medium.

10 randomly selected observation fields, containing 500–1,000 cells were analysed for cell fate and cell death analysis. The expression frequency of selected cell type markers was determined in three independent experiments.

## Results

### EAE disease courses of selected animals

**Figure 1:**
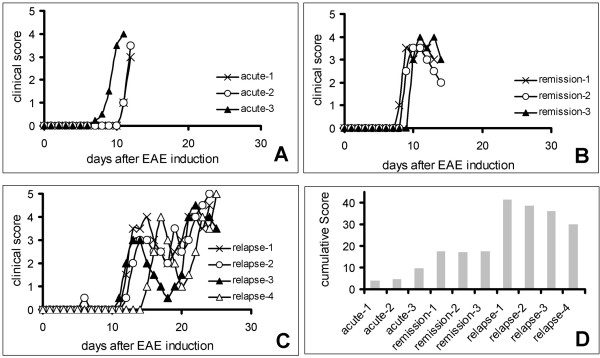
Summary of the clinical courses and cumulative scores of the DA rats chosen for the microarray expression study. A) acute phase, B) recovery phase, C) relapsing phase, D) cumulative disease scores (area under the curve) of the examined animals.

The intention of the study was to compare the spinal cord gene expression profile at three different clinical stages of EAE with the transcriptome of naïve rats. Therefore, female DA rats immunised with recombinant MOG-protein were sacrificed during (1) the acute phase of EAE defined as the first EAE attack leading to a clinical score of at least three (n = 3, days 11 and 12), (2) the recovery phase, i.e. the first day at which the rats began to gain weight after the acute phase (n = 3, days 13 and 14), and (3) the relapsing phase, *i.e*. during a second acute exacerbation after temporary recovery (n = 4, days 24 and 25) (Figure 1). In addition three untreated, healthy control rats were used.

### Spinal cord gene expression in MOG-induced EAE of DA rats

Affymetrix oligonucleotide microarrays representing the complete rat genome were used in this study to characterise the EAE gene expression profile. 14,754 of the 26,000 probe sets were judged as present in at least 20% of the samples by the dchip2006 software [17] and were included in the subsequent analyses.

**Figure 2:**
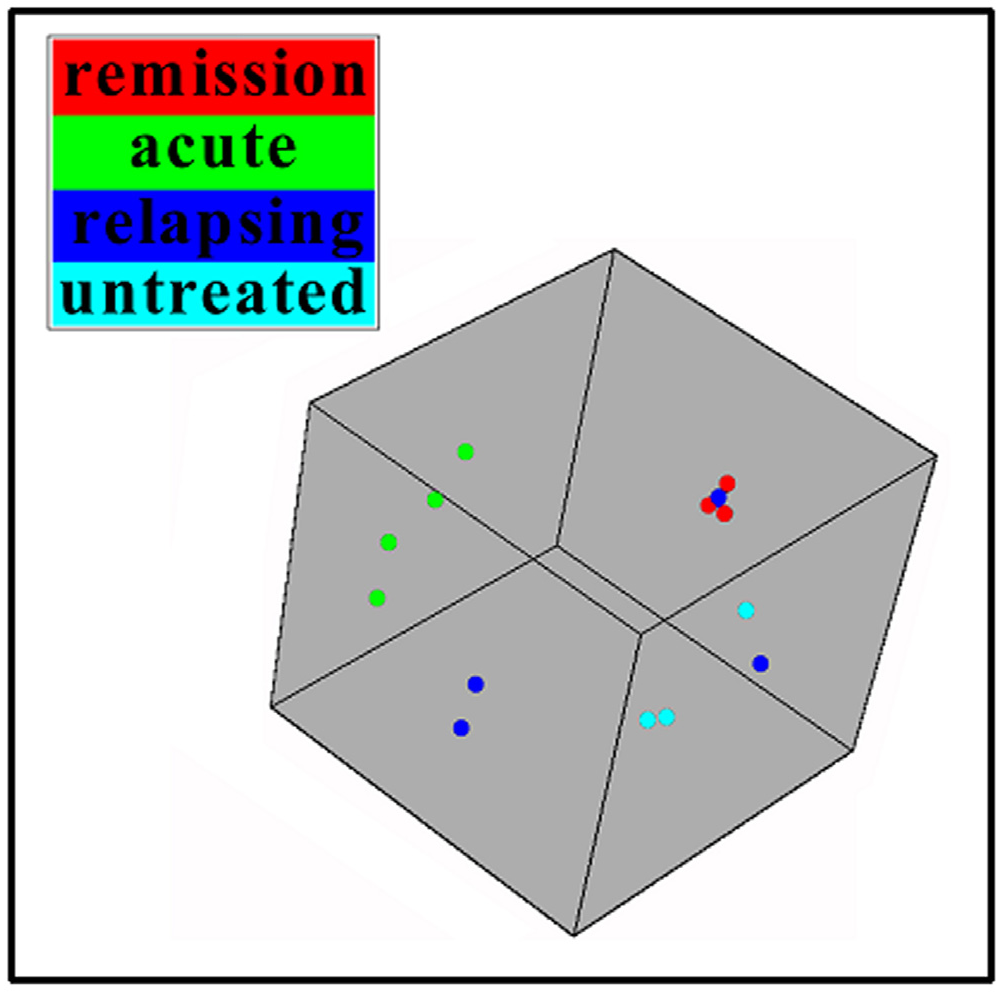
Principal component analysis of the RG U34A hybridizations performed with BRB Array Tools. Hybridization patterns from healthy rats and from rats in the acute and the recovery phase form clusters. This is not the case for hybridization patterns from rats in the relapsing phase.

According to our selection criteria (signal ratio ≥ 2 & p-value ≤ 0.01) 1,165 significantly regulated probe sets were identified. During the acute disease phase 499 probe sets were differentially regulated, during the recovery phase 731 and only 200 in the relapsing phase (Table 2). A principal component analysis of the samples revealed that the hybridization patterns of the rat group sacrificed in the relapsing phase were more heterogeneous than the other groups examined (Figure 2). The heterogeneity of pathogenic events during the relapsing phase may explain the lower number of genes detected as significantly regulated in that group.

**Table 2:** Quantity of genes regulated in particular disease phase compared to healthy rats. Gene expression profiling was carried out using the RG U34 A, B and C microarrays. Regulation was assessed according to defined criteria. All numbered probe sets were present in at least 20% of the samples.

	**acute – healthy**	**recovery – healthy**	**relapsing – healthy**
**upregulated**			
**2-fold**	187	265	66
**4-fold**	109	91	29
**8-fold**	92	36	23
			
**downregulated**			
**2-fold**	74	245	73
**4-fold**	33	76	8
**8-fold**	7	18	1

**Figure 3:**
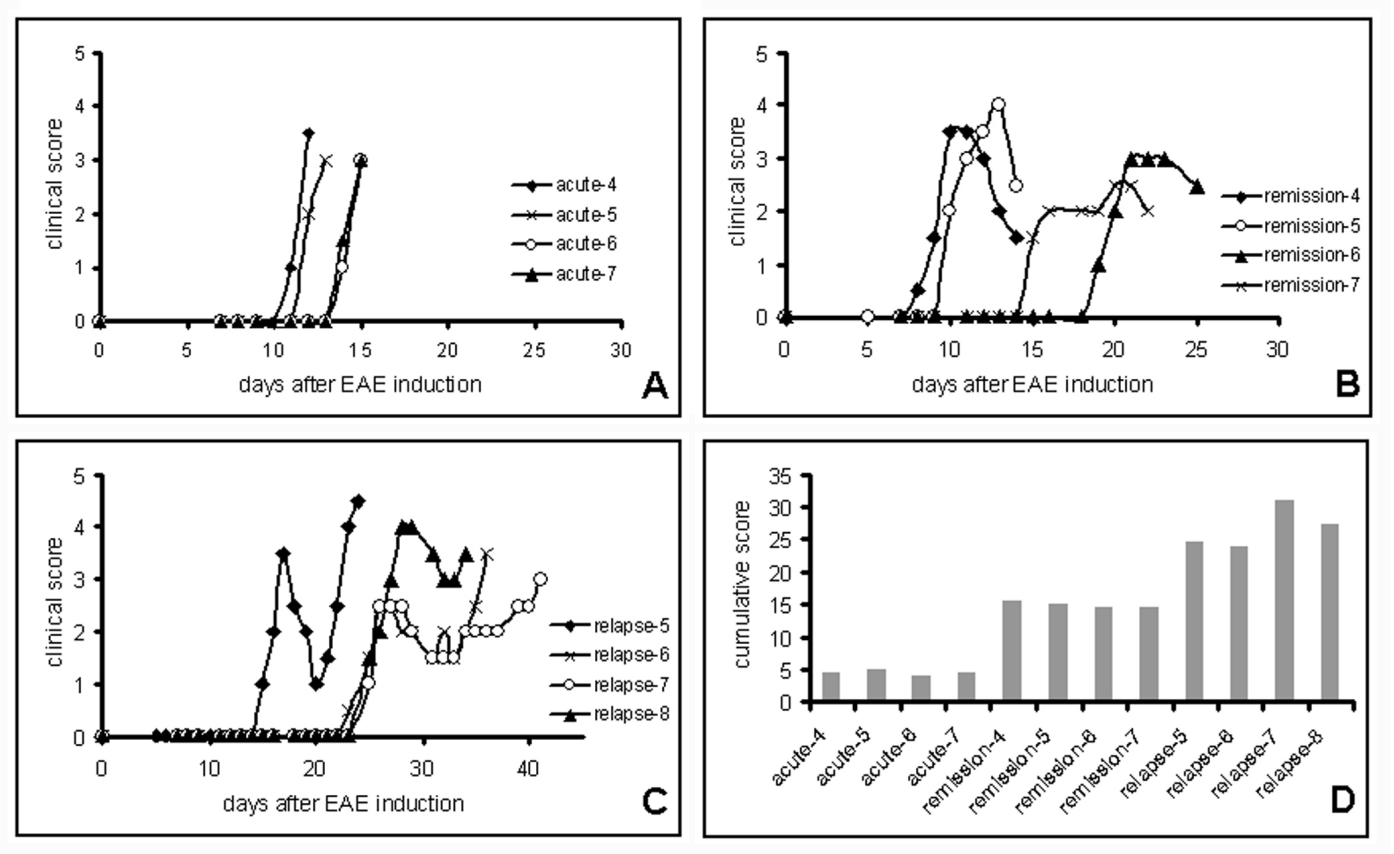
Summary of the clinical courses and cumulative scores of the DA rats chosen for the RealTime-PCR experiments. A) acute phase, B) recovery phase, C) relapsing phase, D) cumulative disease scores (area under the curve) of the examined animals.

**Figure 4:**
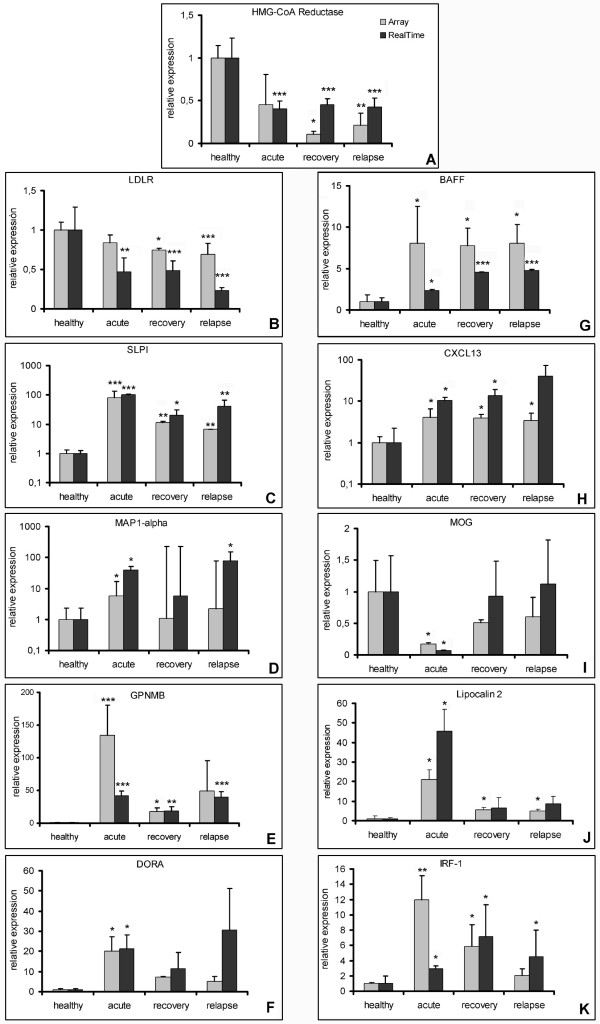
Confirmation of expression values obtained by microarray hybridization with RealTime-PCR values obtained from different samples others than those used for microarray hybridization but with comparable disease course (values are always normalised to quantity of 18S rRNA and presented + std dev, four animals per group) for following genes: A) HMG-CoA Reductase, B) Low density lipoprotein receptor (LDLR), C) Secretory leukocyte protease inhibitor (SLPI), D) MAP1alpha, E) Glycoprotein NMB (GPNMB), F) Downregulated by Activation gene (DORA), G) B cell-activating factor (BAFF), H) CXCL13, I) Myelin-oligodendrocyte-glycoprotein (MOG), J) Lipocalin 2 and K) Interferon-g regulated-factor-1 (IRF-1). The different disease phase were statistically compared using One Way ANOVA analysis of variance (Holm-Sidak method):*:p < 0.05, **: p < 0.01, ***: p < 0.001.

Due to the limited number of repetition of the microarray hybridizations, selected microarray data were validated by quantitative RealTime-PCR using RNA isolated from rats, not used for microarray hybridization, but with identical disease course and similar disease scores as those sacrificed for microarray hybridization (disease courses are in Figure 3). PCR results confirmed that the selected genes were expressed differentially, as detected by the microarrays (Figure 4).

In order to get an estimation of the grade of reliability of our expression data, we compared them with previously published expression data from MS samples or murine EAE. We detected a high number of regulated genes indicating common features of CNS inflammation; *e.g*. increased expression of genes associated with antigen processing [Additional file 1], antigen presentation (Table 3) or of other genes expressed by immune cells [Additional file 2]. Furthermore we detected a suppression of myelin gene expression (Table 4). All four gene groups are regulated in the same manner in MS or in murine EAE models [4-9]. We also found some upregulated transcripts whose relevance for the development of autoimmune inflammation has recently been reported, *e.g*. the induction of BAFF and CXCL13 (Figure 4G &4H) [27], jagged1 [28] and arginase 1 [29], respectively. While confirming the consistency of our data, these results suggest that many pathological processes of MS are reproduced by all EAE models studied so far including EAE in the DA rat as presented here.

**Table 3:** Expression of genes related to antigen presentation within the spinal cord during the disease course of MOG-induced EAE

Probeset	Gene	**healthy**	stddev	**acute**	stddev	**remission**	stddev	**relapsing**	stdev
H33922_f_at	RT1 class Ib gene	**224.7**	35.3	**1085.7**	102.5	**382.8**	88.0	**677.9**	350.6
L40362_f_at		**242.0**	33.1	**913.6**	141.0	**463.3**	84.2	**523.5**	250.4
M11094_g_at		**50.0**	9.2	**182.4**	36.4	**117.8**	13.4	**114.7**	32.7
M11071_f_at		**919.4**	157.0	**3552.1**	547.2	**2325.2**	649.9	**2208.1**	612.6
M24026_f_at		**381.6**	52.5	**1476.6**	78.8	**915.2**	135.4	**1016.0**	408.4
M24324_f_at		**420.3**	27.9	**1904.7**	121.0	**1016.5**	195.5	**1277.4**	592.5
M31018_f_at		**484.4**	56.8	**2383.8**	392.3	**1554.6**	336.0	**1601.8**	614.5
M31038_at		**30.5**	4.2	**180.4**	36.4	**54.4**	4.3	**139.7**	110.0
rc_AA858651_f_at		**386.5**	82.9	**1585.7**	203.2	**887.5**	152.6	**982.4**	295.6
rc_AA945159_s_at		**115.0**	69.6	**502.1**	439.7	**667.2**	69.0	**304.2**	271.3
rc_AI103500_f_at		**290.1**	9.2	**2175.2**	48.2	**513.8**	100.9	**1069.4**	780.4
rc_AI176358_f_at		**299.3**	20.7	**1176.6**	270.5	**611.3**	42.9	**733.8**	256.9
rc_AI235223_at		**213.6**	41.4	**947.6**	95.3	**844.3**	112.6	**714.9**	227.9
AF025308_f_at	MHC class Ib antigen (RT1.Cl)	**127.5**	11.9	**696.2**	190.0	**161.7**	19.6	**413.8**	335.9
L40364_f_at	MHC class I RT1.O type 149 processed pseudogene	**438.8**	41.1	**1100.0**	178.2	**577.9**	87.3	**722.8**	470.2
AF029240_at	MHC class Ib RT1.S3	**21.8**	8.6	**94.2**	23.8	**55.2**	4.2	**35.6**	13.9
AF029240_g_at		**99.5**	9.0	**267.9**	94.7	**185.6**	48.2	**135.9**	50.5
rc_AI235890_s_at		**34.1**	15.5	**228.3**	13.5	**55.6**	21.5	**63.6**	42.4
AF074608mRNA_f_at	MHC class I antigen (RT1.EC2)	**186.2**	21.9	**612.1**	93.6	**493.7**	119.8	**496.0**	116.0
M64795_f_at	MHC class I antigen gene (RT1-u haplotype)	**42.1**	6.0	**322.6**	37.7	**59.5**	13.0	**179.8**	148.6
rc_AI235223_at	RT1 class I, A3	**213.6**	41.4	**947.6**	95.3	**844.3**	112.6	**714.9**	227.9
X57523_at	Transporter 1, ATP-binding cassette, sub-family B (MDR/TAP)	**78.1**	11.6	**292.0**	108.8	**88.2**	24.2	**123.5**	44.1
X57523_g_at		**21.9**	2.9	**308.5**	105.5	**86.4**	31.8	**66.2**	30.7
X63854_at	Transporter 2, ATP-binding cassette, sub-family B (MDR/TAP)	**44.8**	9.5	**125.4**	26.0	**60.6**	1.1	**74.5**	23.5
rc_AI170268_at	Beta-2-microglobulin	**738.0**	270.5	**2559.2**	864.8	**4295.5**	654.1	**2487.2**	1142.1
K02815_s_at	Butyrophilin-like 2 (MHC class II associated)	**86.3**	2.8	**869.9**	155.5	**485.3**	199.1	**519.6**	306.1
M15562_at	MHC class II RT1.u-D-alpha chain mRNA, 3' end	**77.3**	5.9	**1441.2**	355.7	**1088.1**	375.5	**765.1**	244.6
M15562_g_at		**205.6**	126.3	**4436.1**	1998.8	**5944.8**	1630.7	**3230.7**	1453.0
Z49761_at	Major histocompatibility complex, class II, DM alpha	**21.5**	10.3	**343.5**	52.4	**96.9**	5.7	**125.4**	66.4
U31598_s_at		**95.9**	29.7	**799.4**	114.3	**108.6**	12.0	**266.3**	169.8
U31599_at	Major histocompatibility complex, class II, DM beta	**4.5**	1.9	**256.8**	107.7	**22.7**	6.6	**68.0**	63.0
U31599_g_at		**9.9**	2.5	**441.4**	69.5	**69.1**	9.8	**156.8**	116.4
rc_AI171966_at		**42.7**	8.0	**1233.0**	256.7	**433.0**	94.2	**428.3**	286.4
X13044_at	CD74 antigen (invariant polpypeptide of major histocompatibility class II antigen-associated)	**89.2**	22.8	**2866.0**	381.1	**2330.7**	638.0	**1867.9**	218.7
X13044_g_at		**86.5**	22.6	**3206.0**	873.7	**3432.7**	796.6	**2228.7**	364.2
X14254cds_g_at		**73.3**	18.6	**1947.4**	288.9	**1298.5**	384.1	**1417.0**	427.4
X53054_at	MHC class II DLA DRB1 beta chain	**1.9**	0.3	**181.8**	49.4	**99.7**	30.8	**101.5**	28.4
X53054_g_at		**40.3**	7.2	**788.7**	134.2	**745.6**	233.2	**556.7**	157.9
rc_AI045321_at	Class II, major histocompatibility complex, transactivator	**41.0**	11.6	**245.2**	38.0	**124.3**	30.3	**61.7**	21.0
rc_AI228153_s_at	MHC class II antigen RT1.B-1 beta-chain	**25.3**	5.9	**963.5**	320.2	**720.3**	126.6	**451.0**	228.3
X56596_at		**51.0**	18.3	**1064.2**	72.4	**400.0**	84.6	**439.8**	208.1
M36151cds_s_at		**39.1**	6.0	**1090.4**	348.8	**250.9**	68.0	**554.4**	471.8

**Table 4:** Expression of myelin genes within the spinal cord during the disease course of MOG-induced EAE

probe set	Gene	**healthy**	stddev	**acute**	stddev	**recovery**	stddev	**relapsing**	stddev
L21995_s_at	Myelin oligodendrocytic glycoprotein (MOG)	**1082.4**	1035.3	**263.2**	94.2	**249.0**	86.8	**1082.4**	236.7
M99485_at		**903.9**	600.1	**237.2**	83.0	**837.5**	119.2	**903.9**	232.6
K00512_at	Myelin basic Protein (MBP)	**13969.6**	1215.4	**4299.6**	3490.7	**3565.8**	1058.6	**13969.6**	5036.0
rc_AI145512_at		**14362.3**	1660.6	**7131.6**	5155.8	**15716.6**	2812.9	**14362.3**	1822.9
D28111_at	Myelin-associated oligodendrocytic basic Protein (MOBP)	**2241.1**	1428.6	**481.0**	142.3	**989.1**	175.2	**2241.1**	659.3
D28111_g_at		**5595.3**	2369.3	**1488.9**	441.9	**3420.5**	521.4	**5595.3**	819.3
M22357_at	Myelin-associated glycoprotein (MAG)	**294.1**	130.8	**142.0**	26.3	**133.4**	16.7	**294.1**	65.4
M22357_g_at		**2076.6**	1079.9	**922.9**	146.0	**1314.2**	296.9	**2076.6**	251.1
M25888_at	Proteolipid Protein (PLP)	**6608.0**	2018.9	**2179.8**	3490.7	**7643.4**	1983.5	**6608.0**	762.7
rc_AI070277_s_at		**9475.6**	1072.8	**2179.8**	679.7	**6841.0**	963.3	**9475.6**	1407.0
rc_AI072770_s_at		**5556.7**	1923.6	**1176.3**	515.1	**2646.7**	183.7	**5556.7**	2321.2
rc_AA964584_at	Oligodendrocyte myelin glycoprotein (Omg)	**765.4**	185.1	**210.8**	132.7	**680.3**	47.4	**765.4**	195.1
L16532_at	2'-3'-cyclic nucleotide Phosphodiesterase 1 (CNP)	**4482.2**	2061.3	**1168.1**	305.5	**3147.5**	391.2	**4482.2**	2408.6

### Determination of EAE-response CNS genes

Transcripts expressed in the spinal cord of EAE rats are derived either from CNS-resident cells or from infiltrating leukocytes. In order to focus on the regulation of genes expressed by CNS-resident cells and by activated infiltrating cells ("EAE-response CNS genes"), we subtracted genes with expression detectable in lymphoid tissue from the complete list of genes expressed in the spinal cord.

Genes presumably expressed by non-activated infiltrating leukocytes were identified in parallel experiments by hybridization of Affymetrix microarrays with RNA from inguinal lymph nodes of untreated DA rats (n = 5). Subtraction resulted in a list of ~1,500 transcripts of  EAE-response CNS genes expressed in the spinal cord in at least one of  the examined disease phases. This included 184 transcripts differentially expressed during the disease course according to the selection criteria (fold change > 2 & p-value < 0.01, Table 5). According to a hierarchical clustering performed with dchip2006, 29 of  these transcripts showed a marked upregulation during the acute phase [Additional file 3]. Several among these transcripts, notably glycoprotein 49B1, alanyl aminopeptidase (CD13) and lipocalin 2, while not expressed in the lymph nodes of naïve DA rats, are reportedly associated with various activated leukocyte populations [30-32]. This indicates that our subtraction algorithm indeed identified transcripts not constitutively found in naïve or non-activated lymphoid cells but up-regulated upon activation.

**Table 5:** Numbers of "EAE-response CNS genes" regulated in particular disease phase compared to healthy rats. Differentially regulated genes were considered to be "EAE-response CNS genes", when they were not expressed in lymph nodes (based on own microarray experiments).

	**acute – healthy**	**recovery – healthy**	**relapsing – healthy**
**upregulated**			
**2-fold**	8	21	6
**4-fold**	8	5	2
**8-fold**	3	3	0
**downregulated**			
**2-fold**	42	59	34
**4-fold**	20	15	2
**8-fold**	7	4	1

A large group of genes expressed by CNS-resident cells was suppressed during all examined disease phases. This included transcripts encoding for ion channel and transporter proteins (*e.g*., Slc12a2, Slc24a2, Slco1c1 or Cplx1) and regulators of synaptic transmission (e.g. Gabbr1, Ache, Syn2, Reep1, Magi2, Snap25 or Sh3gl2). These findings suggest a profound and sustained impairment of neuronal signal transmission during the course of EAE. The persistent suppression of transcripts for MOG, myelin basic protein (MBP), myelin-associated oligodendrocytic basic protein (MAG) and oligodendrocyte-myelin glycoprotein (OMG) indicates dysfunction or even loss of oligodendrocytes, putative primary targets of the immune response in EAE (Table 4).

Upregulation of CNS genes in the recovery phase but not in the acute phase suggests a potential contribution to repair processes [4]. Twelve CNS-specific genes showed this expression profile (Table 6). Some of these genes had been described to be involved in repair or regenerative processes within the CNS before (Map2c (Microtubule-associated protein 2) [33], Id4 (Inhibitor of DNA binding 4) [34], Ednrb (Endothelin receptor B) [35], Cdh22 (Cadherin 22) [36] and Glrx2 (Glutaredoxin 2) [37]. Interestingly, however, the angiogenic growth factor Pdgfd (Platelet-derived growth factor D (also called spinal cord-derived growth factor B)) [38], Pnlip (Pancreatic lipase), the cytokinesis controlling armadillo protein Pkp4 (Plakophilin 4) [39], the peroxysome regulator Pex11a (Peroxisome biogenesis factor 11a) [40] and Hoxb8 (Homeobox B8) have not been associated with repair processes in the CNS yet. Two transcripts of this group are not yet defined (Genbank accession: AI070489 and AI229198).

**Table 6:** "EAE-response CNS transcripts" associated with the recovery phase and possibly contributing to repair processes. Summary of the ratios of the expression values of the genes in the particular disease phase and healthy rats. For all genes, the differences between any disease phase and healthy rats are statistically significant according to the defined selection criteria

Probe set	Gene Title	acute/healthy	recovery/healthy	relapsing/healthy
rc_AI229198_at	Transcribed locus	0.15	1.88	1.05
rc_AI237207_at	Inhibitor of DNA binding 4	0.22	3.23	0.93
X17682_s_at	microtubule-associated protein 2	0.38	1.73	0.98
rc_AI070489_at	Transcribed locus	0.48	2.69	0.84
X57764_s_at	endothelin receptor type B	0.54	3.41	1.40
AA848702_at	Platelet-derived growth factor, D	0.54	2.11	0.97
rc_AI101014_at	Glutaredoxin 2 (thioltransferase)	0.59	2.28	1.20
S65355_at	endothelin receptor type B	0.62	3.89	1.59
M58369_at	pancreatic lipase	0.80	5.10	3.84
rc_AA892296_at	homeo box B8 (mapped)	0.81	2.14	1.29
rc_AA892128_at	peroxisomal biogenesis factor 11A	0.96	2.01	1.38
rc_AA957117_at	Cadherin 22	1.09	2.99	1.44
rc_AA964481_at	Plakophilin 4	1.18	2.22	1.45

#### The cholesterol biosynthesis pathway in EAE

Cholesterol is an essential component of eukaryotic plasma membranes and represents around 30% of the lipid content within the CNS. In the mature CNS, the highest cholesterol content can be found in oligodendrocyte myelin. Cholesterol availability in oligodendrocytes is rate-limiting for myelination [41].

**Figure 5:**
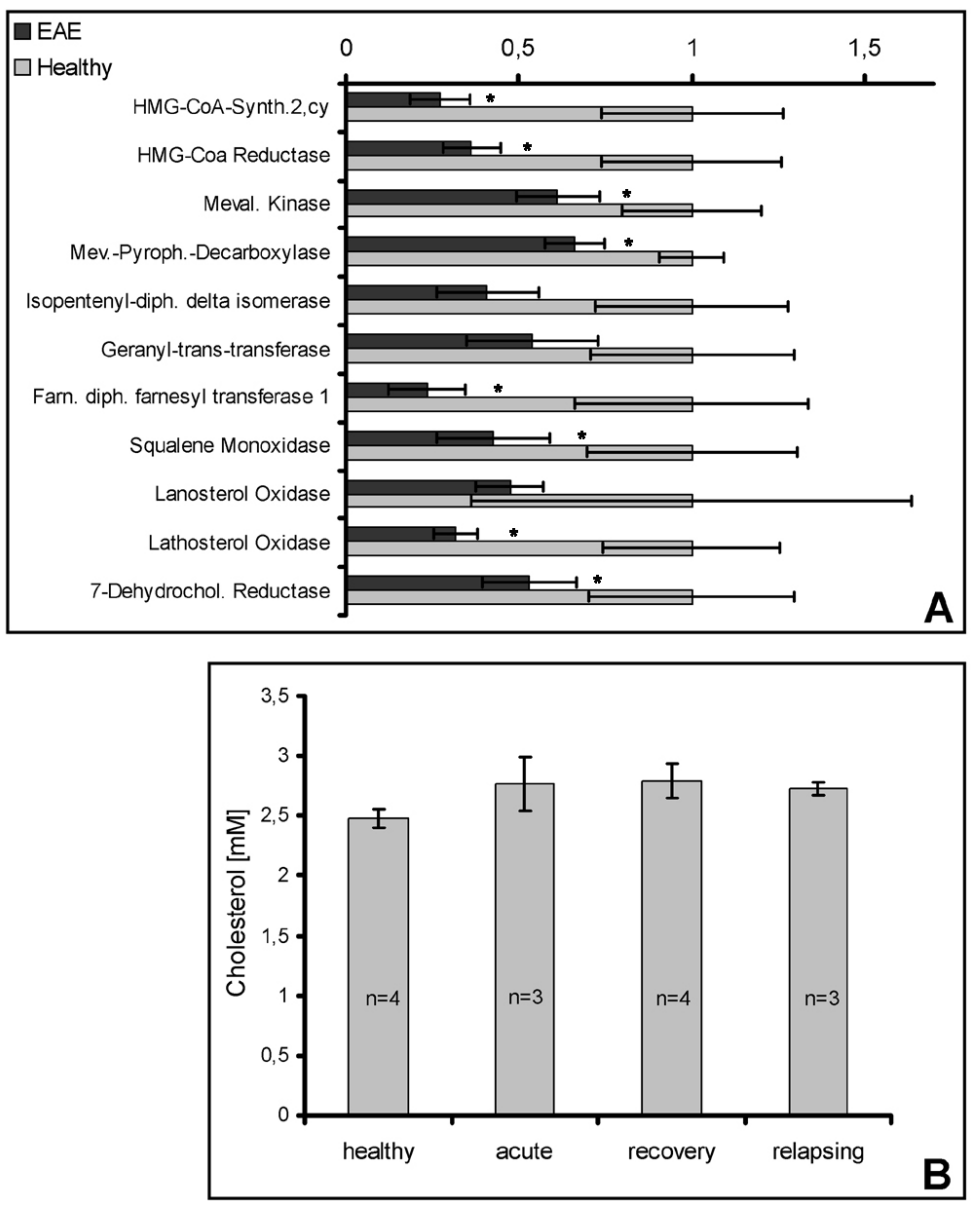
**Downregulation of the cholesterol biosynthesis pathway during EAE**. **A: **Summary of the average expression values of genes of the cholesterol biosynthesis pathway (± std dev). As we only noticed minor differences of the expression of these genes within the observed disease phases, the expression values obtained for the acute, recovery and relapsing disease phase were merged to one value representing the average expression of the particular gene during EAE. All values are correlated to the expression values of the corresponding gene in the spinal cord of healthy rats. The EAE gene expression was compared to the expression within healthy rats using a paired t test. *: p < 0.05. B: Average cholesterol concentration in spinal cord extracts. Rats from distinct disease phase were sacrificed, their spinal cord dissected and lipids and lipoproteins extracted by RIPA buffer. Cholesterol concentrations were determined as described in Material and Methods.

Interestingly, our data revealed a suppression of many genes associated with the cholesterol biosynthesis pathway throughout the acute, recovery and relapse time points (Figure 5A, Table 7). This suppression included HMG-CoA (3-**h**ydroxy-3-**m**ethyl**g**lutaryl-**CoA**) reductase catalysing the rate limiting step in cholesterol biosynthesis (Figure 4A).

**Table 7:** Summary of the expression of genes of the cholesterol biosynthesis within the spinal cord during EAE

Affymetrix ID	Gene Title	Gene Symbol	mean healthy	mean acute	mean recovery	mean relapsing
U36992_at	cytochrome P450, subfamily 7B, polypeptide 1	Cyp7b1	98.59	195.80	276.35	162.86
rc_AI171090_at	3-hydroxy-3-methylglutaryl CoA lyase	Hmgcl	202.20	227.46	239.53	261.08
rc_AI171090_g_at			54.98	105.97	67.11	72.45
rc_AA945052_at			102.85	198.26	202.62	199.80
M33648_at	3-hydroxy-3-methylglutaryl-Coenzyme A synthase 2	Hmgcs2	138.44	148.71	172.73	176.42
M33648_g_at			53.88	71.08	127.08	89.43
M29472_at	mevalonate kinase	Mvk	579.91	347.12	365.24	334.64
rc_AA924198_s_at			89.59	30.12	42.34	41.04
D45252_s_at	2,3-oxidosqualene: lanosterol cyclase	Lss	133.77	96.43	52.29	62.71
U31352_at			78.55	43.51	25.09	30.56
U53706_at	Di-P-Mevalonate Decarboxylase	Mvd	337.31	275.95	170.16	249.65
rc_AI236566_at	Geranyl-trans-transferase	Gtt	539.00	251.35	431.77	336.83
rc_AI180442_at			73.18	30.33	24.05	30.71
M95591_at	farnesyl diphosphate farnesyl transferase 1	Fdft1	361.69	140.36	10.93	57.25
M95591_g_at			177.78	63.88	14.50	37.18
rc_AA859392_s_at			150.44	60.54	12.54	29.40
rc_AI229016_s_at			683.89	363.56	292.72	350.46
D37920_at	squalene monoxidase	Sqle	454.41	196.60	217.42	194.23
rc_AA819300_at			106.60	47.75	40.46	44.46
AF003835_at	isopentenyl-diphosphate delta isomerase	Idi1	358.66	107.23	75.43	70.95
rc_AI236611_at			70.34	40.75	43.74	49.80
M29249cds_at	3-hydroxy-3-methylglutaryl-Coenzyme A reductase	Hmgcr	88.42	27.52	2.90	10.18
rc_AA924210_at			274.91	136.54	79.63	86.09
X55286_at			29.11	11.75	1.63	5.22
X55286_g_at			49.36	24.45	7.98	12.30
AB016800_at	7-dehydrocholesterol reductase	Dhcr7	173.32	60.96	86.66	80.03
AB016800_g_at			179.32	65.09	120.92	104.75
rc_AI177004_s_at	3-hydroxy-3-methylglutaryl-Coenzyme A synthase 1	Hmgcs1	741.89	171.63	86.91	96.25
X52625_at			1524.92	577.65	626.34	493.05
rc_AI043855_at	sterol-C5-desaturase (fungal ERG3, delta-5-desaturase)-like	Sc5d	435.08	141.91	79.75	124.68
AB004096_at	cytochrome P450, subfamily 51	Cyp51	246.53	77.01	100.46	87.21

A comparable general decrease of the expression of cholesterol transport proteins was not noticed (Table 8). Rather, apolipoprotein C I (ApoC1) and CD36 were strongly upregulated during the acute disease phase. The expression profile and the known expression pattern of CD36 suggest that its upregulation is probably caused by the immigration of CD36-expressing macrophages into the spinal cord during the acute phase. The expression of the cholesterol carrier apolipoprotein E (ApoE) was increased during the recovery disease phase. The only cholesterol transport genes showing a comparable expression decrease during EAE as the genes of the cholesterol biosynthesis were the Glutamate oxaloacetate transaminase 2, the Low density lipoprotein receptor (LDLR, Figure 4B) and the Oxysterol binding protein-like proteins 1A and 9.

**Table 8:** Genes of cholesterol transport proteins being expressed in at least 20% of the samples

Affymetrix ID	Gene	**healthy**	stdev	**acute**	stdev	**recovery**	stdev	**relapsing**	stdev
M27440_at	Apolipoprotein B	**49.73**	46.6	**15.96**	4.4	**67.62**	12.3	**70.33**	45.3
rc_AA997806_at		**53.94**	35.0	**34.14**	14.0	**126.63**	89.0	**50.86**	15.7
X15512_at	Apolipoprotein C-I	**15.92**	10.9	**415.98**	167.1	**92.05**	18.6	**316.06**	346.8
X55572_at	Apolipoprotein D	**2297.39**	322.6	**1723.52**	84.7	**1839.49**	283.5	**2001.28**	476.4
X04979_at	Apolipoprotein E	**2980.67**	301.3	**5192.22**	3005.3	**8207.48**	1115.9	**5531.19**	2185.4
rc_AI179131_at	ATP-binding cassette, sub-family G, member 1	**298.63**	46.0	**522.68**	92.8	**362.14**	43.9	**497.34**	188.2
AF063302mRNA#3_s_at	Carnitine palmitoyltransferase 1b	**83.97**	5.2	**65.47**	18.4	**69.39**	15.5	**81.62**	21.0
AF072411_at	CD36 antigen	**11.01**	4.9	**180.95**	119.4	**2.73**	0.3	**7.13**	10.9
AF072411_g_at		**27.84**	17.9	**458.91**	256.2	**16.00**	2.3	**26.43**	23.7
rc_AA946368_at		**25.01**	16.7	**226.76**	62.5	**37.79**	11.8	**20.09**	15.0
U23407_at	Cellular retinoic acid binding protein 2	**45.99**	10.2	**65.57**	39.9	**32.39**	8.5	**53.95**	19.5
U89529_at	Fatty acid transport protein	**347.54**	64.0	**242.31**	9.3	**219.71**	31.5	**275.59**	87.1
M18467_at	Glutamate oxaloacetate transaminase 2	**511.64**	35.4	**439.99**	23.1	**259.87**	46.9	**285.07**	47.0
rc_AA892012_g_at		**49.39**	14.7	**50.71**	27.6	**13.64**	9.4	**48.43**	29.0
L32132_at	Lipopolysaccharide binding protein	**12.60**	4.6	**111.01**	24.1	**40.52**	9.4	**48.87**	25.2
rc_AI043724_at	Low density lipoprotein receptor	**337.57**	35.9	**229.21**	39.8	**166.04**	17.8	**163.08**	36.8
rc_AI070411_at		**121.75**	44.2	**91.50**	49.1	**7.31**	5.7	**41.72**	24.8
rc_AI070135_at	Oxysterol binding protein-like 1A	**206.55**	78.6	**90.59**	15.9	**80.88**	3.6	**122.10**	54.7
rc_AI070882_at		**336.07**	149.6	**173.52**	52.9	**208.84**	28.7	**262.69**	76.8
rc_AI137224_at		**243.29**	43.5	**111.30**	25.0	**107.49**	16.8	**155.86**	52.6
AF040261_s_at	phosphatidylcholine transfer protein	**26.58**	5.9	**34.18**	2.0	**29.38**	2.5	**30.49**	13.4
rc_AA901035_at	Oxysterol binding protein-like 6	**106.57**	7.3	**93.54**	17.1	**105.03**	5.6	**83.83**	14.7
rc_AA944269_at	STARD3 N-terminal like	**458.93**	116.8	**363.80**	51.7	**696.50**	62.4	**467.24**	130.5
rc_AI234872_at	Oxysterol binding protein-like 9	**262.14**	11.2	**274.79**	70.0	**165.72**	71.9	**191.55**	10.3
rc_AI237694_at		**32.46**	5.4	**33.46**	11.2	**13.17**	3.1	**21.90**	5.1
rc_AI070093_at	Oxysterol binding protein-like 5	**328.76**	41.3	**232.18**	16.7	**228.50**	17.1	**210.83**	67.5
rc_AA892260_g_at	Steroidogenic acute regulatory protein	**39.59**	4.7	**70.89**	11.8	**49.75**	4.3	**52.25**	10.0
M58287_s_at	Sterol carrier protein 2, liver	**13.39**	6.1	**9.38**	1.1	**9.96**	1.1	**15.50**	2.4
M62763complete_seq_at		**209.95**	25.1	**145.84**	38.6	**171.01**	8.9	**204.37**	52.6
rc_AI171674_at	Very low density lipoprotein receptor	**89.35**	15.8	**43.11**	15.5	**98.31**	7.1	**89.84**	23.9

As the transcription of the HMG-CoA reductase or LDLR is inhibited by the presence of cholesterol derivatives, we assessed the total cholesterol concentration in the spinal cord tissue and detected no change of the cholesterol concentration at any time point of EAE (Figure 5B). Hence, the downregulation of the expression of the enzymes of the cholesterol biosynthesis pathway was not associated with a corresponding decrease of cholesterol concentration (Table 8).

#### SLPI in MOG-induced EAE of DA rats

**Figure 6:**
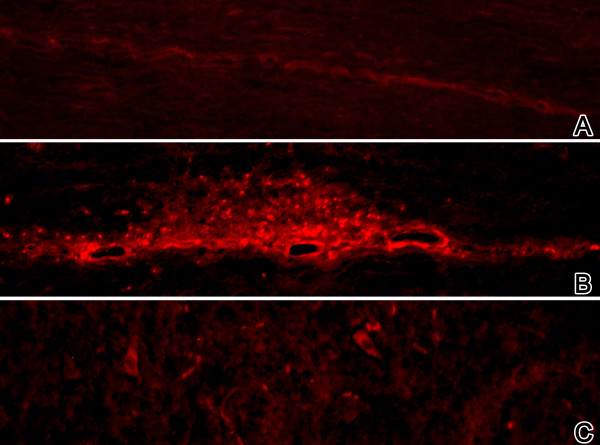
**SLPI protein is expressed in the spinal cord during the acute and the relapsing phase. SLPI-staining of longitudinal spinal cord slices obtained from DA rats**. **A: **healthy animal, **B: **acute phase (score 3.5) and **C: **relapsing phase (score 3.5), (Magnification 50x).

The expression of the secretory leukocyte protease inhibitor (SLPI) was approximately 100-fold upregulated during the acute disease phase and more than 10-fold during the recovery and relapsing phases (Figure 4C). Immunohistochemical analyses of spinal cord slices from rats suffering from acute EAE showed a strong SLPI staining restricted to the inflammatory infiltrates. During the relapsing phase the expression pattern of SLPI was more scattered and therefore less associated with inflammatory infiltrates (Figure 6). In spinal cord slices from rats sacrificed during the recovery phase only background staining was seen.

**Figure 7:**
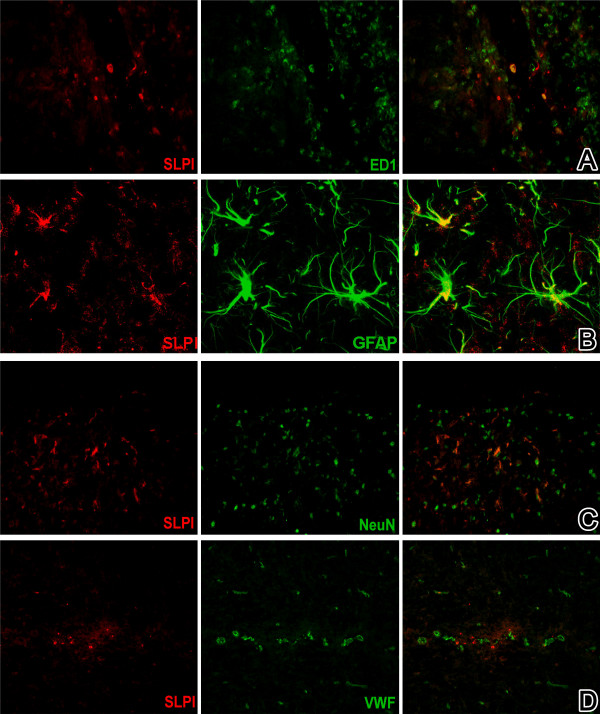
SLPI expression within the spinal cord is associated with ED1-expressing macrophages or activated microglia in the acute phase (score 3.5, A) and with GFAP-positive astrocytes (B) and NeuN expressing neuronal cells (C) especially during the relapsing phase (score 3.5); there was no association of SLPI with von-Willebrand-factor positive endothelial cells (D). Magnification: 100x.

To characterise the cell types expressing SLPI in the CNS, we also performed immunohistochemical studies. During the acute phase of EAE cells SLPI staining mostly colocalised with ED1-positive cells, *i.e*. macrophages and activated microglia within inflammatory infiltrates (Figure 7). In the recovery phase, we additionally detected SLPI in a high proportion of GFAP-positive astrocytes as well as in sporadic NeuN positive neurons (Figure 7). We did not see costaining with the endothelial cell marker von-Willebrand-factor at any time point.

**Figure 8:**
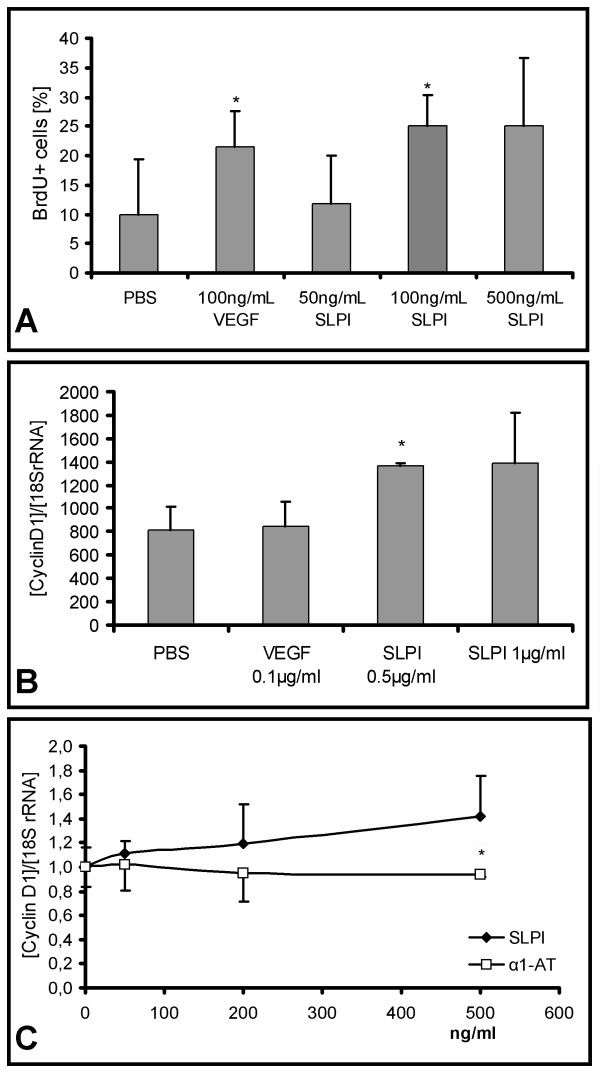
**SLPI promotes proliferation of adult neural stem cells and induces cyclin D1**. **A: **Proliferation of rat neural stem cells after treatment with SLPI or VEGF, respectively. Cells were treated with indicated amounts of SLPI or VEGF for three days. Afterwards, they were pulsed with 10 μM BrdU. Proportion of BrdU-positive cells (+ std dev) was determined with the FITC BrdU Flow Kit. Presentation of a representative result of three experiments. *:p < 0.05 (according to a One Way ANOVA analysis of variance (Holm-Sidak method). **B: **RealTime-PCR assessment of cyclin D1 expression (n = 3, + std dev) in rat adult stem cells after an incubation period of three days with the indicated amounts of SLPI or VEGF, respectively. **C: **Determination of cyclin D1 expression (n = 3, + std dev) of adult neural stem cells treated for three days with indicated amounts of SLPI or α1-AT. Presentation of a representative result of three experiments.*:p < 0.05 (according to a One Way ANOVA analysis of variance (Holm-Sidak method)).

**Figure 9:**
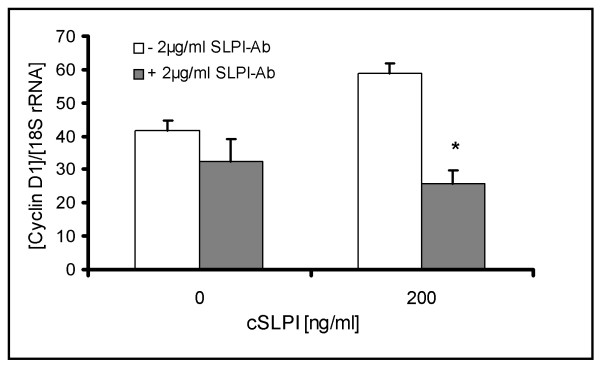
**Confirmation of specificity of SLPI's effects on NSCs**. Determination of cyclin D1 expression (+ std dev) of adult neural stem cells treated for three days with indicated amounts of SLPI with or without 2 μg/ml SLPI. *: p < 0.05 referred to the difference of cyclin D1 expression of cultures incubated with or without SLPI antibodies (according to a paired t test).

To determine whether the increased SLPI expression within the spinal cord might contribute to regenerative processes, we incubated multipotent neural stem cells (NSCs) from the subventricular zone of adult Wistar rats [25] for up to seven days in growth medium with varying amounts of SLPI. This resulted in a reproducible augmentation of cell proliferation by more than 100%, quantified by both cell counting and determination of BrdU-positive cells. SLPI-mediated enhancement of proliferation was similar to the well established effect of VEGF on proliferation [42] (Figure 8A). As SLPI has been reported to induce the cell cycle regulator cyclin D1 [43], we assessed its expression in NSC treated with SLPI by RealTime-PCR. Indeed, we found cyclin D1 upregulation in parallel and concomitant with the increase of proliferation when NSC were incubated with 0.5 to 1 μg/ml SLPI (Figure 8B). SLPI antibodies could prevent the cyclin D1 induction caused by recombinant SLPI confirming the specificity of the effects of recombinant SLPI (Figure 9).

We then compared SLPI's effects on neural stem cells with those of the protease inhibitor α_1_-antitrypsin (α_1_-AT). Some of the features of SLPI are paralleled by α_1_-AT, *e.g*. it induces the hepatocyte growth factor [44] and inhibits the chemotaxis of neutrophils [45] as well as acute inflammatory responses [46]. Interestingly, α_1_-AT did not induce the expression of cyclin D1 (Figure 8C), thereby suggesting that the promotion of cell proliferation cannot only be attributed to SLPI's protease inhibiting activity.

**Figure 10:**
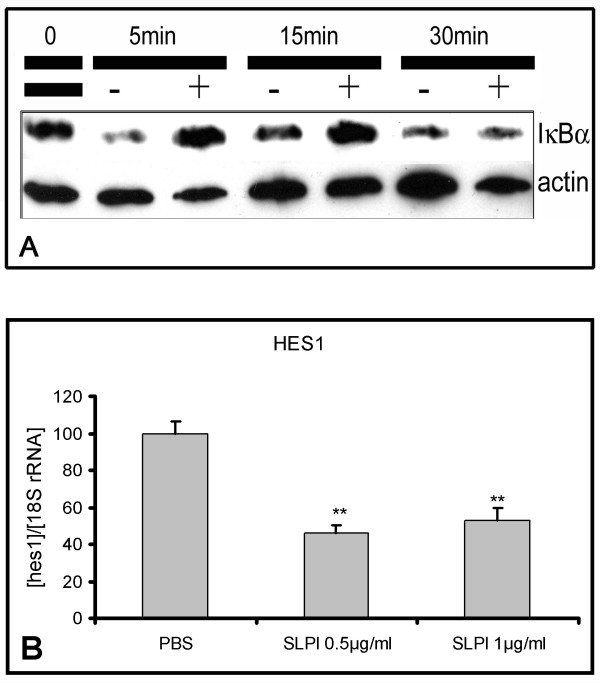
**SLPI prevents TNFα-induced IκBα degradation and suppresses HES1**. **A: **Western Blot for IκBα with protein extracts from neural stem cells stimulated for the indicated periods with 10 ng/ml TNFα with (+) or without (-) 500 ng/ml SLPI. Presentation of one of two experiments. **B: **RealTime-PCR assessment of HES1 expression (n = 3, + std dev) in rat adult NSC after an incubation period of three days with the indicated amounts of SLPI and consecutive four days in differentiation medium. Presentation of a representative result of three experiments. *:p < 0.05, **: p < 0.01 (according to a One Way ANOVA analysis of variance (Holm-Sidak method)).

SLPI inhibits the degradation of IκBα in monocytes thereby promoting its accumulation within the cell [47]. We observed a reduction of TNFα-induced IκBα degradation by SLPI in NSC (Figure 10A). By binding to the promoter of the cell differentiation suppressor HES1 (**H**airy/**e**nhancer of **s**plit, drosophila, homolog of, **1**) IκBα represses its expression independent of NFκB [48]. We determined the expression of the Notch-target gene HES1 in NSCs treated again with SLPI and detected a significant downregulation of HES1 mRNA in the SLPI cultures (Figure 10B).

**Figure 11:**
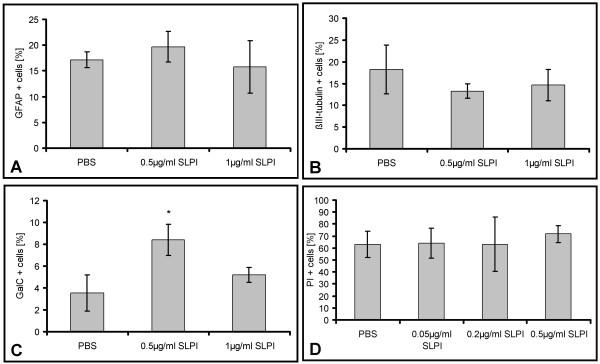
**SLPI enhances the differentiation of oligodendroglial cells. **Differentiation and cell death analysis of NSC treated with indicated amounts of SLPI. NSC mounted on microscope slides were cultivated for three days with the specified amounts of SLPI in growth medium and subsequently for seven days in differentiation medium. Cells were stained for cell specific markers and counted. The shown values are representative for three independent experiments. **A: **proportion of GFAP positive astroglial cells (± std dev), **B: **proportion of βIII-tubulin positive neuronal cells (± std dev), **C: **proportion of GalC expressing oligodendrocytes (± std dev). *: p < 0.05 (according to a One Way ANOVA analysis of variance (Holm-Sidak method)). **D: **To detect dying cells, 50 μg/ml propidium iodide was added to the culture medium. After ten minutes of incubation. The fraction of PI positive cells and the total cell number was determined by counting. Presentation of the proportion of dying cells and total cell number (± std dev).

As HES1 inhibits the differentiation of NSCs [49], we finally asked whether SLPI influences the cell fate of the NSCs. After incubating NSCs for three days with SLPI in growth medium and another seven days in differentiation medium, we noticed almost no change of the number of GFAP expressing astrocytes and of βIII-tubulin expressing neurons, but a significant dose-dependent increase of oligodendrocytes expressing GalC. SLPI did not influence the survival of NSCs as indicated by a propidium iodide staining (Figure 11). This implies that the SLPI-mediated increase of Gal-C-positive cells was not caused by increased survival or decreased cell death.

## Discussion

In this study we examined the transcriptional profile of mRNA isolated from spinal cord tissue of MOG-induced EAE in DA rats. Comparable approaches have been adopted in several murine EAE models before (*e.g*. [4,6,8,9,18,19,50]). In contrast to prior studies, however, we selected a chronic rat EAE model for a genome-wide analysis of the CNS expression profile. We compared the transcriptomes of the acute, recovery and relapsing phases of EAE with the transcriptome of untreated, healthy rats and with each other to detect new EAE-associated genes and elucidate their relative importance for disease processes, in particular recovery and repair. More than 1,100 differentially expressed genes were identified. To overcome the statistical problems caused be the analysis of only three animals per disease phase, we performed quantitative RealTime-PCRs for selected targets using rats which have not been sacrificed for microarray hybridizations. Importantly, the microarray data were generally validated by RealTime-PCR.

Common features of CNS inflammation were observed, confirming data from other EAE-models and providing an external validation of our analyses, *e.g*. the upregulation of genes associated with protein catabolism and antigen presentation [6,20], the downregulation of oligodendrocyte-specific genes [20,51] or of genes involved in synaptic transmission [4].

By subtracting transcripts of "naïve LNC" *in silico *from the total list of regulated genes we were able to focus on "EAE-response CNS genes". This group includes transcripts differentially regulated in CNS-resident cells [4] and genes *de novo *induced in populations of activated immigrated leukocytes. As a consequence, this algorithm does not reveal genes constitutively expressed in LNC and up- or downregulated at the time of disease.

We identified 12 genes which were upregulated in the recovery phase but not during the acute phase. The exclusive expression of a gene during the recovery phase may implicate a role for the corresponding gene product in regeneration. Indeed, five among these genes have already been linked to neuroprotective or repair processes within the CNS, albeit not in EAE. For example, glutaredoxin 2 protects neuronal cells *in vitro *from death by excitotoxicity [37]. The expression of map2c might be associated with developing neurons and with nerve cell survival during stress [33]. Id4 is an important factor for neuronal differentiation [34] and has been described as an oligodendrocyte differentiation inhibitor during maturation [52]. Id4 might therefore contribute to the block of oligodendrocyte differentiation known to be operative in autoimmune CNS inflammation. These molecules and of the other transcripts shown in Table 6 warrant further investigation. Interestingly, plakophilin 4 (p0071) influences the activity of presenilin 1 [53] that in turn controls the activity of notch receptors contributing to the inhibition of the maturation of oligodendroglial progenitor cells in EAE [54].

The downregulation of virtually all genes of the cholesterol biosynthesis during lesion evolution of autoimmune CNS inflammation has not been described previously. So far, only the suppression of HMG-CoA reductase and of squalene monooxidase has been reported, occurring in inflammatory lesions of more than 75% of MS patients [20]. Of note, the expression of genes involved in extra- and intracellular transport of cholesterol was not affected in a similar manner, largely excluding sustained damage of cholesterol synthesising cells within the CNS. Our findings might indicate a feedback inhibition of cholesterol synthesis, because the overall cholesterol concentration in the spinal cord remained unchanged during all stages of EAE. The transcription of many genes of the cholesterol biosynthesis pathway, including the HMG-CoA Reductase and LDL receptor is negatively regulated by the presence of cholesterol or its derivatives [55,56]. Both molecules have been found to be suppressed in our study. The downregulation of the genes of the cholesterol biosynthesis pathway including the HMG-CoA Reductase might be of special interest, because HMG-CoA Reductase Inhibitors (Statins) are assumed to ameliorate the MS disease by modulating *e.g*. the activity of T cells and of microglial cells or by enhancing the survival of oligodendroglial progenitor cells [57].

The secretory leukocyte protease inhibitor (SLPI) is an 11.7 kDa protein originally identified in parotid gland secretions, in seminal fluid, and in cervical, nasal and bronchial mucous. Later it was also found in human neutrophils, peritoneal macrophages, and in astrocytes and neurons under ischemic conditions [14]. SLPI has been recognised as a potent inhibitor of leukocyte serine proteases, including elastase and cathepsin G from neutrophils, chymase and tryptase from mast cells, as well as trypsin and chymotrypsin from pancreatic acinar cells, respectively [58]. In addition, SLPI suppresses bacterial growth [59] and inhibits HIV-1 infection of macrophages [60]. By inhibiting the degradation of IκB, SLPI appears to exert anti-inflammatory functions on macrophages, neutrophils and B cells [15,61]. SLPI reduces inflammatory gene expression and diminishes inflammatory cell accumulation after hepatic and lung injuries [62,63].

Notably, SLPI also promotes the proliferation of epithelial cells [43] and of haematopoietic stem cells [64]. Mice deficient in SLPI show impaired cutaneous wound healing with increased inflammation and TGF-beta activity, as well as increased elastase activity [65]. The expression of SLPI is highly upregulated within ischemic brain tissue, where it has been ascribed a neuroprotective role, possibly because of rapid inhibition of activated proteases and its suppression of inflammatory responses [14].

In this study, microarray and RealTime-PCR analyses revealed SLPI to be the most strongly induced gene within the spinal cord during EAE. Using immunohistochemistry we detected a strong staining for SLPI protein in association with perivascular infiltrates. In accordance with findings reported for ischemic brain tissue [14], we detected SLPI protein in neurons and astrocytes, but found it also colocalised with markers for activated macrophages or microglial cells.

We asked whether the SLPI overexpression in the spinal cord during EAE might support cell renewal in the CNS. Interestingly, SLPI induced and increased proliferation of adult NSCs, associated with the selective induction of the growth-promoting factor cyclin D1. The latter effect is probably not caused solely by SLPI's inhibitory action on proteases, because α_1_-antitrypsin, a comparable protease inhibitor [46], did not cause a similar upregulation of cyclin D1. Rather, the inhibition of the IκBα degradation by SLPI [66] and its occupancy of NFκB binding sites [61], resulting in diminished activity of NFκB, may provide an explanation for our observation. This hypothesis is further supported by the inhibition of TNFα-induced IκBα degradation and the suppression of the cell cycle regulator HES1 in SLPI treated NSC cultures. IκBα suppresses the expression of HES1 independently of NFκB by binding to the HES1 promoter [48]. HES1 suppression, however, is expected to enhance differentiation of neural stem cells [67].

Interestingly, SLPI treatment promoted dose-dependently the differentiation of NSCs towards Gal-C expressing oligodendrocytes. We did not detect population-specific differences in cell survival, thereby excluding a selective survival advantage of differentiating oligodendrocyte precursors. The dose-dependency of SLPI's effects have been described before and explained by opposing effects of SLPI's direct promoter activity and its inhibition of the nuclear translocation of NFκB [43,66]. It is rather tempting to envisage SLPI involved in remyelination by promoting the maturation of progenitor cells towards mature myelinating cells. Furthermore, by inducing factors like HGF (hepatocyte growth factor) SLPI might also act as a chemoattracting factor guiding NSC to the lesions [44,68]. These questions will be addressed in forthcoming studies.

## Conclusion

We identified novel features of gene expression in the CNS during EAE, in particular the suppression of genes of cholesterol biosynthesis and a strong upregulation of a gene not previously associated with autoimmune inflammation in the CNS but with potential relevance for both control of inflammation and tissue destruction as well as promotion of tissue repair. We suggest that SLPI, by promoting the proliferation of adult NSCs and their differentiation towards oligodendroglial cells, may contribute to repair processes *in vivo*, expanding its functional spectrum beyond protease inhibition, prevention of tissue damage and modulation of inflammation.

## Competing interests

The authors declare that they have no competing interests.

## Authors' contributions

AMM conceived the design of the study, performed experiments as well as experimental analysis and prepared the manuscript. XP contributed to the study design and manuscript preparation and performed experiments, TS performed microarray hybridization, supported the data analysis and aided in the preparation of the manuscript, IK revised the manuscript critically, LA and SCD provided the neural stem cells and revised the manuscript, GG conceived the study design and provided critical analysis of the manuscript and AS aided in the study design, helped to draft the manuscript and provided useful suggestions. All authors read and approved the final version of the manuscript.

## Supplementary Material

Additional file 1 Expression of genes related to protein catabolism during the disease course of MOG-induced EAE Click here for file

Additional file 2 Expression of immune response genes during the disease course of MOG-induced EAE Click here for file

Additional file 3 Overview of "EAE-response CNS genes". Presentation of the normalised signal intensities of the "EAE-response CNS genes". For all genes, the differences between any disease phase and healthy rats are statistically significant according to the defined selection criteria. Click here for file
