# Supplementary material for: Novel role for SLPI in MOG-induced EAE revealed by spinal cord expression analysis
Source: J Neuroinflammation. 2008 May 26;5:20. doi: 10.1186/1742-2094-5-20 (PMC2438345; doi:10.1186/1742-2094-5-20)
Supplement: Additional file 1 — Expression of genes related to protein catabolism during the disease course of MOG-induced EAE [file 1742-2094-5-20-S1.doc]

| Probeset | Gene | **healthy** | Stddev | **acute** | Stddev | **remission** | Stddev | **relapsing** | Stddev |
| --- | --- | --- | --- | --- | --- | --- | --- | --- | --- |
| Z48444cds_at | Proteasome subunit, alpha type 1 (Psma1) | **139.5** | 13.9 | **215.6** | 14.3 | **119.6** | 22.8 | **133.7** | 10.0 |
| E03358cds_at | Proteasome subunit, alpha type 2 (Psma2) | **284.8** | 8.3 | **362.4** | 78.4 | **173.4** | 7.7 | **227.8** | 67.1 |
| rc_AI170403_at |  | **314.5** | 150.5 | **414.1** | 158.6 | **712.4** | 117.0 | **480.9** | 295.5 |
| rc_AI171738_at | Proteasome subunit, alpha type 3 (Psma3) | **23.9** | 4.6 | **25.6** | 9.0 | **25.8** | 3.8 | **34.7** | 13.6 |
| rc_AI169479_s_at | Proteasome subunit, alpha type 5 (Psma5) | **426.8** | 51.7 | **730.0** | 98.4 | **331.0** | 41.1 | **435.7** | 94.4 |
| D10756_at |  | **101.8** | 8.7 | **166.7** | 36.7 | **67.1** | 13.0 | **103.2** | 35.4 |
| D10756_g_at |  | **250.0** | 18.7 | **358.8** | 54.4 | **363.4** | 38.1 | **322.3** | 68.6 |
| rc_AI112633_s_at | Proteasome subunit, alpha type 6 (Psma6) | **183.3** | 9.0 | **418.6** | 87.1 | **76.6** | 7.9 | **145.0** | 50.9 |
| D10755_s_at |  | **583.9** | 65.5 | **823.3** | 131.2 | **780.0** | 29.7 | **766.9** | 244.5 |
| rc_AI071208_at |  | **88.1** | 36.0 | **132.2** | 11.3 | **147.8** | 6.7 | **110.6** | 46.3 |
| D30804_at | Proteasome subunit, alpha type 7 (Psma7) | **302.3** | 42.7 | **353.4** | 22.4 | **292.7** | 5.1 | **338.0** | 57.7 |
| D30804_g_at |  | **411.1** | 90.9 | **588.4** | 88.3 | **796.4** | 72.2 | **537.5** | 230.3 |
| rc_AA849722_at | Proteasome subunit, beta type 1 (Psmb1) | **352.2** | 42.7 | **450.8** | 35.8 | **403.1** | 70.6 | **358.6** | 78.1 |
| D21799_g_at | Proteasome subunit, beta type 2 (Psmb2) | **339.3** | 47.0 | **527.7** | 76.0 | **307.4** | 78.8 | **374.1** | 44.3 |
| D21799_at |  | **131.0** | 10.9 | **183.2** | 27.4 | **71.4** | 17.1 | **141.3** | 53.4 |
| D21800_g_at | Proteasome subunit, beta type 3 (Psmb3) | **250.4** | 14.7 | **353.8** | 66.7 | **266.4** | 12.0 | **310.1** | 61.4 |
| D21800_at |  | **233.1** | 35.0 | **232.6** | 23.5 | **171.2** | 19.1 | **228.5** | 46.6 |
| rc_AI172162_at | Proteasome subunit, beta type 4 (Psmb4) | **279.4** | 65.6 | **501.8** | 158.8 | **541.9** | 33.3 | **395.8** | 118.7 |
| L17127_at |  | **523.5** | 71.3 | **448.7** | 284.5 | **500.4** | 246.5 | **512.1** | 264.0 |
| L17127_g_at |  | **477.8** | 57.9 | **504.6** | 208.9 | **565.4** | 220.0 | **461.2** | 155.8 |
| D45247_at | Proteasome subunit, beta type 5 (Psmb5) | **543.1** | 153.6 | **463.2** | 123.4 | **166.7** | 8.2 | **425.9** | 210.0 |
| D45247_g_at |  | **712.5** | 59.5 | **730.6** | 56.9 | **812.8** | 68.6 | **723.3** | 135.7 |
| rc_AA891226_s_at |  | **425.4** | 87.2 | **409.5** | 130.0 | **207.3** | 27.8 | **330.5** | 117.8 |
| rc_AI011563_s_at |  | **270.9** | 108.3 | **264.9** | 110.0 | **37.7** | 4.8 | **229.5** | 141.5 |
| D10754_at | Proteasome subunit, beta type 6 (Psmb6) | **173.4** | 32.7 | **164.4** | 29.9 | **64.5** | 7.9 | **123.8** | 49.6 |
| D10754_g_at |  | **208.8** | 32.7 | **257.2** | 1.0 | **165.5** | 21.3 | **212.2** | 37.6 |
| rc_AI104300_at | Proteasome subunit, beta type 7 (Psmb7) | **613.6** | 76.1 | **603.7** | 196.7 | **400.3** | 22.2 | **537.9** | 173.8 |
| rc_AI178629_s_at | Proteasome subunit, beta type 8 (Psmb8) | **29.8** | 18.7 | **364.0** | 102.5 | **156.8** | 34.6 | **117.0** | 33.6 |
| D10729_s_at |  | **65.4** | 5.8 | **436.0** | 67.7 | **110.5** | 26.9 | **162.6** | 104.1 |
| D10757_at | Proteasome subunit, beta type 9 (Psmb9) | **3.0** | 0.9 | **190.3** | 32.7 | **19.4** | 13.9 | **24.6** | 20.1 |
| D10757_g_at |  | **25.4** | 7.9 | **413.4** | 23.0 | **141.8** | 44.0 | **73.8** | 27.1 |
| rc_AI012340_s_at |  | **73.6** | 23.1 | **895.4** | 128.3 | **195.1** | 83.5 | **172.2** | 89.4 |
| rc_AI177481_s_at |  | **16.8** | 8.5 | **317.4** | 99.6 | **54.3** | 12.5 | **62.8** | 54.7 |
| D45249_at | protease (prosome, macropain) 28 subunit, alpha | **583.1** | 102.0 | **1118.2** | 131.8 | **411.1** | 119.0 | **670.9** | 221.5 |
| D45249_g_at |  | **259.4** | 17.4 | **1052.8** | 180.1 | **619.6** | 138.2 | **564.1** | 82.8 |
| rc_AA848487_s_at |  | **231.9** | 99.9 | **1538.9** | 145.1 | **356.8** | 65.7 | **514.7** | 285.0 |
| rc_AI011981_at | protease (prosome, macropain) 28 subunit, beta | **46.3** | 22.2 | **65.1** | 39.3 | **45.3** | 8.0 | **59.6** | 11.1 |
| rc_AI011981_g_at | protease (prosome, macropain) 28 subunit, beta | **360.5** | 68.3 | **2094.3** | 221.9 | **740.0** | 327.8 | **490.5** | 108.9 |
| rc_AA851169_s_at |  | **293.0** | 66.8 | **1332.3** | 187.4 | **373.2** | 83.8 | **373.9** | 95.6 |
| D45250_s_at |  | **110.7** | 18.7 | **413.0** | 126.6 | **96.0** | 5.3 | **87.6** | 36.2 |
| D50696_at | Proteasome (prosome, macropain) 26S subunit, ATPase 1 (Psmc1) | **399.6** | 42.7 | **284.3** | 28.7 | **274.8** | 28.7 | **281.0** | 61.8 |
| U13895_s_at | Proteasome (prosome, macropain) 26S subunit, ATPase 2 (Psmc2) | **144.9** | 48.9 | **111.1** | 30.1 | **30.7** | 5.0 | **105.3** | 52.7 |
| D50694_at |  | **253.0** | 42.4 | **237.6** | 32.6 | **168.5** | 24.3 | **187.1** | 30.7 |
| U77918_at | Proteasome (prosome, macropain) 26S subunit, ATPase 3 (Psmc3) | **153.5** | 28.0 | **168.5** | 19.8 | **79.5** | 5.5 | **125.5** | 43.8 |
| D50695_at | Proteasome (prosome, macropain) 26S subunit, ATPase 4 (Psmc4) | **143.5** | 14.2 | **158.3** | 33.2 | **105.0** | 10.5 | **135.5** | 36.9 |
| rc_AA859869_s_at | Proteasome (prosome, macropain) 26S subunit, non-ATPase, 1 (Psmd1) | **314.8** | 31.8 | **325.3** | 21.8 | **301.1** | 10.5 | **240.0** | 47.8 |
| AJ006340_s_at |  | **145.4** | 17.8 | **123.2** | 7.3 | **119.0** | 11.7 | **97.3** | 21.6 |
| U57050_g_at |  | **505.0** | 41.9 | **567.0** | 86.0 | **595.3** | 87.2 | **601.1** | 103.5 |
| rc_AA799887_s_at | Proteasome (prosome, macropain) 26S subunit, non-ATPase, 4 (Psmd4) | **175.8** | 16.4 | **198.0** | 31.1 | **134.9** | 9.2 | **129.8** | 18.3 |
| rc_AI236731_at |  | **199.2** | 20.1 | **192.9** | 54.1 | **164.0** | 50.9 | **163.5** | 17.0 |
| AB017188_at |  | **164.7** | 44.0 | **139.1** | 54.1 | **91.2** | 13.9 | **130.6** | 48.2 |
| AB017188_g_at |  | **361.0** | 107.2 | **345.4** | 56.2 | **279.0** | 48.2 | **272.3** | 75.3 |
| rc_AI175576_at | Proteasome (prosome, macropain) 26S subunit, non-ATPase, 9 (Psmd9) | **180.7** | 5.8 | **144.5** | 28.0 | **179.3** | 25.1 | **167.1** | 37.0 |
| rc_AA957491_at | Proteasome (prosome, macropain) 26S subunit, non-ATPase, 10 (Psmd10) | **241.2** | 9.8 | **188.7** | 14.9 | **306.1** | 29.3 | **266.2** | 77.0 |
| rc_AI172453_at |  | **233.6** | 22.8 | **165.6** | 10.2 | **324.8** | 38.0 | **252.2** | 124.4 |
| AB022014_at |  | **35.1** | 2.7 | **26.3** | 8.5 | **22.4** | 7.6 | **30.6** | 10.6 |
| X05300_at | Ripopherin I (Rpn1) | **154.3** | 27.3 | **186.3** | 23.1 | **141.9** | 15.2 | **152.9** | 37.0 |
| X55298_at | Ripopherin II (Rpn2) | **189.2** | 4.9 | **355.6** | 52.5 | **180.9** | 19.5 | **194.8** | 38.2 |
| M37584_at | H2A histone family, member Z (H2AZ) | **117.4** | 36.0 | **353.7** | 97.6 | **397.2** | 9.7 | **243.6** | 114.2 |
| D17296_at | Ubiquitin C | **1649.6** | 85.3 | **1486.5** | 259.9 | **1722.8** | 247.3 | **1608.6** | 355.2 |
| rc_AA799612_at | Ubiquitin conjugating enzyme E2b (Ube2b) | **236.7** | 6.7 | **190.5** | 17.2 | **179.8** | 4.3 | **192.2** | 9.3 |
| M62388_at |  | **164.3** | 3.0 | **142.5** | 40.8 | **75.7** | 15.7 | **137.8** | 55.7 |
| M62388_g_at | Ubiquitin conjugating enzyme E2b (Ube2b) | **61.7** | 7.0 | **66.7** | 12.4 | **56.6** | 14.9 | **76.3** | 18.3 |
| rc_AI233359_at | Ubiquitin conjugating enzyme E2-17kDa 4 (Ube2d2) | **232.1** | 24.3 | **145.0** | 29.7 | **188.0** | 24.0 | **177.4** | 55.1 |
| rc_AI104247_at |  | **133.3** | 8.6 | **138.0** | 7.9 | **106.9** | 22.5 | **105.4** | 7.7 |
| U13176_at |  | **100.5** | 9.5 | **122.2** | 36.1 | **58.1** | 4.9 | **74.6** | 43.0 |
| AF099093_at | Ubiquitin conjugating enzyme E2 G1 (Ube2g1) | **81.2** | 32.2 | **57.2** | 21.3 | **42.0** | 0.7 | **60.1** | 20.8 |
| AF099093_g_at |  | **61.0** | 24.5 | **38.7** | 20.2 | **21.2** | 6.2 | **35.7** | 16.0 |
| rc_AA851376_at |  | **528.6** | 140.5 | **488.9** | 82.7 | **1102.6** | 184.3 | **721.8** | 289.2 |
| rc_AA996896_at |  | **27.6** | 2.6 | **36.2** | 7.5 | **42.5** | 4.1 | **34.0** | 11.1 |
| rc_AI229984_at |  | **18.3** | 8.9 | **18.9** | 10.8 | **47.7** | 10.3 | **24.3** | 16.8 |
| rc_AA925340_at | Ubiquitin conjugating enzyme E2N (Ube2n)  Cathepsin B | **407.9** | 42.4 | **662.9** | 75.4 | **785.1** | 93.8 | **530.6** | 187.2 |
| X82396_at |  | **557.1** | 74.3 | **2373.7** | 182.8 | **845.4** | 14.8 | **1387.0** | 831.3 |
| rc_AA926360_at |  | **32.2** | 8.7 | **720.8** | 62.1 | **163.7** | 21.4 | **153.3** | 60.9 |
| D90404_at |  | **19.2** | 5.4 | **643.2** | 86.7 | **47.3** | 7.8 | **173.2** | 183.7 |
| D90404_g_at |  | **23.5** | 4.1 | **499.1** | 117.3 | **117.7** | 22.0 | **103.7** | 37.1 |
| X54467_at | Cathepsin D | **348.5** | 45.5 | **1019.7** | 123.6 | **517.6** | 35.3 | **1146.1** | 912.2 |
| rc_AI235585_s_at |  | **325.9** | 76.3 | **817.8** | 150.9 | **332.2** | 85.2 | **863.8** | 728.8 |
| D45187_s_at | Cathepsin E | **18.5** | 17.2 | **33.4** | 12.4 | **60.5** | 5.0 | **61.4** | 29.6 |
| M38135_at | Cathepsin H | **128.1** | 12.4 | **270.1** | 81.0 | **314.3** | 80.7 | **350.1** | 104.9 |
| rc_AA925246_at | Cathepsin K | **68.9** | 65.4 | **268.5** | 68.4 | **258.9** | 19.7 | **243.3** | 201.6 |
| rc_AI176595_s_at | Cathepsin L | **328.7** | 55.0 | **693.4** | 132.3 | **457.3** | 13.1 | **671.2** | 216.6 |
| rc_AI230591_at | Cathepsin L2 (Precursor Y,U) | **12.3** | 7.5 | **73.5** | 8.7 | **20.1** | 4.4 | **31.7** | 14.4 |
| rc_AI237380_at |  | **8.6** | 4.8 | **42.5** | 8.6 | **14.3** | 3.0 | **28.6** | 14.4 |
| L03201_at | Cathepsin S | **1043.6** | 41.5 | **3704.3** | 1347.7 | **5266.8** | 716.1 | **4033.5** | 1445.6 |
| rc_AA818798_at | Cathepsin Z | **95.4** | 7.6 | **861.7** | 153.0 | **282.6** | 31.2 | **499.2** | 458.7 |
| rc_AA851184_s_at |  | **114.3** | 29.3 | **1767.4** | 326.8 | **518.1** | 64.5 | **707.3** | 604.4 |
| rc_AA852018_at | Interferon gamma inducible protein 30 | **217.8** | 18.9 | **1985.3** | 493.2 | **873.8** | 43.2 | **933.5** | 380.5 |
| rc_AA925353_at | Lysosomal-associated protein transmembrane 5 (Laptm5) | **263.9** | 100.6 | **870.7** | 164.1 | **365.2** | 25.9 | **687.1** | 524.2 |
| rc_AI234133_at |  | **98.2** | 17.4 | **621.8** | 73.1 | **210.6** | 21.2 | **382.4** | 258.4 |
| L11319_at | Signal peptidase complex 18kD | **59.0** | 10.0 | **101.3** | 35.0 | **38.8** | 5.4 | **83.7** | 41.9 |
| L11319_g_at |  | **116.1** | 12.5 | **238.1** | 78.3 | **86.1** | 4.8 | **164.7** | 91.7 |
| rc_AI172497_at | Peptide/histidine transporter PHT2 | **54.2** | 7.5 | **261.5** | 65.3 | **105.7** | 9.5 | **159.6** | 98.4 |
| AF098301_at | Neural F box protein NFB42 | **378.0** | 40.5 | **178.1** | 14.9 | **233.5** | 40.1 | **283.0** | 43.5 |
| rc_AA859882_s_at | Ubiquitin carboxy-terminal hydrolase L1 | **1531.2** | 45.9 | **525.7** | 81.4 | **941.4** | 180.0 | **835.2** | 298.9 |
| D10699_at |  | **1257.4** | 140.4 | **322.8** | 47.8 | **387.3** | 34.9 | **667.1** | 312.8 |
| D10699_g_at |  | **828.5** | 38.7 | **235.6** | 21.1 | **219.7** | 36.0 | **447.9** | 211.2 |
