# Supplementary material for: Novel role for SLPI in MOG-induced EAE revealed by spinal cord expression analysis
Source: J Neuroinflammation. 2008 May 26;5:20. doi: 10.1186/1742-2094-5-20 (PMC2438345; doi:10.1186/1742-2094-5-20)
Supplement: Additional file 2 — Expression of immune response genes during the disease course of MOG-induced EAE [file 1742-2094-5-20-S2.doc]

| Affymetrix ID | Gene | **healthy** | stddev | **acute** | stddev | **recovery** | stddev | **relaps.** | stddev |
| --- | --- | --- | --- | --- | --- | --- | --- | --- | --- |
| U22414_at | chemokine (C-C motif) ligand 3 | **1.97** | 0.4 | **147.99** | 147.8 | **19.76** | 5.0 | **21.11** | 1 |
| X17053mRNA_s_at | chemokine (C-C motif) ligand 2 | **76.52** | 65.3 | **3275.62** | 725.7 | **810.09** | 163.8 | **227.67** | 98.9 |
| X17053cds_s_at |  | **193.04** | 5.2 | **1805.1** | 1042.0 | **223.73** | 8.1 | **215.74** | 42.2 |
| AJ223184_at | immunoglobulin superfamily, member 6 | **2.54** | 0.9 | **76.58** | 34.8 | **28.40** | 1.4 | **20.23** | 10.3 |
| AJ223184_g_at |  | **8.66** | 3.8 | **86.14** | 5.5 | **27.44** | 2.8 | **20.93** | 6.7 |
| rc_AI012235_at | chemokine (C-X-C motif) ligand 11 | **30.72** | 2.9 | **819.99** | 318.9 | **263.27** | 111.0 | **53.17** | 18.2 |
| M98820_at | interleukin 1 beta | **48.39** | 3.1 | **705.35** | 498.5 | **132.66** | 22.7 | **70.05** | 12.1 |
| M98820_g_at |  | **2.73** | 0.4 | **120.11** | 100.6 | **18.63** | 6.48 | **17.88** | 3.9 |
| E01884cds_s_at |  | **2.77** | 1.5 | **73.26** | 61.3 | **4.44** | 1.3 | **3.67** | 0.8 |
| M80367_at | guanylate nucleotide binding protein 2 | **4.89** | 1.1 | **127.93** | 35.6 | **80.33** | 35.6 | **21.35** | 11.3 |
| X73371_at | Fc receptor, IgG, low affinity IIb | **27.63** | 8.3 | **699.99** | 160.6 | **214.30** | 3.7 | **273.26** | 98.1 |
| M32062_at | Fc receptor, IgG, low affinity III | **87.90** | 14.9 | **1925.20** | 284.5 | **662.98** | 223.3 | **852.01** | 540.9 |
| M32062_g_at |  | **150.41** | 17.9 | **2491.65** | 298.8 | **761.64** | 189.6 | **975.52** | 696.6 |
| E13732cds_at | chemokine (C-C motif) receptor 1 | **12.30** | 6.1 | **238.64** | 134.7 | **3.98** | 2.6 | **13.62** | 14.1 |
| X05111_at | CD2 antigen | **1.66** | 0.8 | **30.07** | 13.2 | **2.67** | 1.7 | **2.75** | 0.7 |
| X60769mRNA_at | CCAAT/enhancer binding protein (C/EBP), beta | **73.48** | 11.4 | **1261.39** | 281.1 | **407.45** | 108.2 | **250.28** | 94.7 |
| M34253_g_at | interferon regulatory factor 1 | **158.60** | 25.5 | **2548.07** | 577.1 | **1873.57** | 781.3 | **550.21** | 239.6 |
| rc_AI011757_at | Fc fragment of IgG, low affinity IIIa, receptor | **45.54** | 8.5 | **726.54** | 231.6 | **226.39** | 47.8 | **303.17** | 204.7 |
| rc_AA924105_at | chemokine (C-C motif) ligand 6 | **41.97** | 24.2 | **611.83** | 114.6 | **118.52** | 15.0 | **180.45** | 93.2 |
| M26744_at | interleukin 6 | **2.03** | 1.1 | **26.73** | 17.6 | **8.58** | 4.7 | **1.96** | 0.7 |
| X71127_g_at | complement component 1, q subcomponent | **98.38** | 35.7 | **1293.83** | 543.3 | **1192.91** | 197.7 | **2441.12** | 1367.8 |
| AF087943_s_at | CD14 antigen | **115.11** | 9.4 | **1496.6** | 447.0 | **251.24** | 33.1 | **243.93** | 97.1 |
| AF087944mRNA_s_at |  | **216.01** | 23.1 | **1208.8** | 553.8 | **164.16** | 24.4 | **265.06** | 120.6 |
| rc_AA901350_at | Guanylate nucleotide binding protein 4 (predicted) | **91.02** | 12.9 | **1048.75** | 135.1 | **548.90** | 171.5 | **229.64** | 97.4 |
| rc_AI045440_at | sialophorin | **5.00** | 2.6 | **57.19** | 26.8 | **29.83** | 11.2 | **14.19** | 8.0 |
| AF053312_s_at | chemokine (C-C motif) ligand 20 | **18.66** | 7.1 | **209.75** | 87.6 | **23.57** | 6.0 | **22.15** | 8.0 |
| U17035_s_at | chemokine (C-X-C motif) ligand 10 | **19.60** | 8.3 | **191.93** | 86.9 | **104.49** | 28.4 | **51.90** | 49.2 |
| U10894_s_at | allograft inflammatory factor 1 | **96.61** | 9.6 | **941.50** | 163.9 | **608.78** | 151.7 | **684.88** | 254.4 |
| U17919_s_at |  | **181.66** | 15.8 | **1124.75** | 117.6 | **566.05** | 161.2 | **786.77** | 361.9 |
| rc_AI010262_at | interleukin 4 receptor, alpha | **36.67** | 3.8 | **348.86** | 89.8 | **60.71** | 10.1 | **74.69** | 29.3 |
| rc_AI060017_at | lymphocyte cytosolic protein 2 | **29.69** | 0.0 | **263.94** | 108.7 | **94.24** | 11.4 | **110.28** | 78.0 |
| rc_AA945737_at | chemokine (C-X-C motif) receptor 4 | **10.34** | 2.7 | **86.65** | 2.7 | **31.66** | 1.7 | **30.68** | 10.4 |
| X71127_at | complement component 1, q subcomponent | **267.92** | 41.7 | **2171.60** | 1242.7 | **2608.84** | 268.5 | **3108.21** | 823.2 |
| rc_AA892506_at | coronin, actin binding protein 1A | **175.59** | 14.6 | **1380.46** | 118.1 | **559.84** | 173.5 | **730.72** | 421.8 |
| rc_AA851245_at | PYD and CARD domain containing | **94.45** | 10.5 | **716.84** | 120.7 | **285.59** | 31.7 | **310.53** | 178.1 |
| D11445exon#1-4_s_at | chemokine (C-X-C motif) ligand 1 | **90.33** | 4.0 | **648.91** | 482.7 | **117.29** | 43.8 | **99.25** | 9.8 |
| Y12009_at | chemokine (C-C motif) receptor 5 | **56.50** | 16.7 | **364.34** | 82.7 | **81.32** | 28.0 | **123.41** | 57.3 |
| rc_AI104781_at | arachidonate 5-lipoxygenase activating protein | **56.22** | 9.7 | **354.29** | 62.3 | **117.87** | 57.5 | **149.13** | 27.1 |
| X52196cds_at |  | **157.52** | 14.2 | **605.41** | 78.9 | **132.58** | 12.1 | **247.61** | 125.5 |
| M58587_at | interleukin 6 receptor, alpha | **74.05** | 8.3 | **431.25** | 170.2 | **415.36** | 64.1 | **387.91** | 290.5 |
| M92059_s_at | complement factor D (adipsin) | **49.70** | 17.1 | **265.76** | 144.3 | **262.61** | 40.3 | **306.68** | 129.9 |
| rc_AI030587_at | tumor necrosis factor receptor superfamily, member 1b | **55.47** | 1.0 | **285.13** | 66.4 | **98.20** | 3.0 | **136.58** | 52.5 |
| rc_AI171962_s_at | annexin A1 | **363.93** | 219.2 | **1777.36** | 409.6 | **660.55** | 18.8 | **601.04** | 256.0 |
| S57478cds_s_at |  | **372.69** | 123.3 | **1410.2** | 415.5 | **343.68** | 29.8 | **430.44** | 183.3 |
| X52498cds_at | transforming growth factor, beta 1 | **160.68** | 17.7 | **753.73** | 405.4 | **247.69** | 37.0 | **484.81** | 378.1 |
| X14319cds_g_at | T-cell receptor beta chain | **41.31** | 2.2 | **185.83** | 42.2 | **42.50** | 4.4 | **66.71** | 26.9 |
| rc_AA891690_g_at | tumor necrosis factor (ligand) superfamily, member 13 | **183.66** | 31.2 | **807.58** | 49.1 | **205.81** | 35.1 | **228.20** | 55.3 |
| rc_AA942958_at | G-protein signalling modulator 3 (AGS3-like, C. elegans) | **111.03** | 30.3 | **476.18** | 174.4 | **247.84** | 12.2 | **308.87** | 105.7 |
| rc_AA996499_at | complement component 1, q subcomponent | **313.43** | 41.3 | **1336.4** | 817.3 | **1896.10** | 82.3 | **3261.66** | 1594.2 |
| U59801_at | integrin alpha M | **84.03** | 19.6 | **336.01** | 114.9 | **50.52** | 7.2 | **181.19** | 152.6 |
| M10072mRNA_s_at | protein tyrosine phosphatase, receptor type, C | **160.51** | 23.0 | **584.37** | 109.6 | **187.85** | 7.0 | **304.24** | 189.2 |
| X52477_at | complement component 3 | **146.22** | 27.9 | **526.06** | 91.3 | **1693.40** | 222.3 | **1316.83** | 266.2 |
| M29866_s_at |  | **195.58** | 46.3 | **690.05** | 115.2 | **1243.53** | 173.2 | **1418.77** | 728.4 |
| M14656_at | secreted phosphoprotein 1 | **1360.63** | 594.4 | **4750.18** | 965.2 | **1688.46** | 337.1 | **2215.09** | 852.1 |
| rc_AA892259_at | Interferon regulatory factor 8 | **209.63** | 13.5 | **731.12** | 105.0 | **493.23** | 88.2 | **494.67** | 222.7 |
| rc_AA899685_at |  | **67.54** | 7.1 | **182.05** | 68.1 | **80.73** | 6.3 | **150.69** | 104.2 |
| U77777_s_at | interleukin 18 | **109.78** | 20.3 | **372.45** | 53.9 | **355.13** | 41.8 | **292.17** | 106.7 |
| rc_AI045144_at | complement component 6 | **29.75** | 0.3 | **100.55** | 70.6 | **48.91** | 5.2 | **60.20** | 31.2 |
| rc_AI044222_at | chemokine (C-X-C motif) ligand 9 | **29.69** | 0.0 | **97.20** | 44.3 | **40.37** | 9.2 | **38.94** | 7.9 |
| AB003042_at | complement component 5, receptor 1 | **385.14** | 29.4 | **1166.54** | 97.8 | **380.68** | 97.5 | **434.93** | 35.2 |
| rc_AI008629_at | exosome component 9 | **51.59** | 1.9 | **154.16** | 7.3 | **75.08** | 6.7 | **60.94** | 10.9 |
| M98049_s_at | pancreatitis-associated protein | **64.55** | 2.2 | **191.65** | 97.0 | **174.50** | 38.4 | **54.60** | 5.7 |
| rc_AA891576_s_at | complement component 1, q subcomponent | **187.13** | 18.9 | **547.84** | 144.2 | **256.44** | 52.1 | **930.91** | 697.0 |
| AF057025_at | toll-like receptor 4 | **15.53** | 3.2 | **44.95** | 6.7 | **46.08** | 6.6 | **35.25** | 9.5 |
| rc_AI044644_at | Chemokine (C-C motif) receptor 5 | **254.86** | 38.6 | **722.22** | 107.3 | **508.58** | 28.2 | **454.99** | 67.4 |
| D00403_g_at | interleukin 1 alpha | **44.45** | 5.6 | **125.66** | 52.4 | **91.64** | 10.6 | **57.29** | 20.0 |
| rc_AA924029_at | phospholipid scramblase 1 | **70.14** | 3.3 | **187.59** | 42.9 | **117.58** | 3.0 | **108.28** | 19.6 |
| D50558_g_at | cd86 antigen | **41.76** | 3.0 | **111.25** | 34.9 | **53.57** | 7.7 | **78.21** | 37.1 |
| M23566exon_s_at | alpha-2-macroglobulin | **748.39** | 238.3 | **1959.20** | 1015.9 | **2799.86** | 70.6 | **2532.19** | 1070.9 |
| D88250_at | complement component 1, s subcomponent | **330.24** | 122.2 | **857.69** | 409.8 | **959.28** | 112.2 | **664.25** | 316.2 |
| rc_AI169104_at | chemokine (C-X-C motif) ligand 4 | **240.67** | 35.2 | **618.70** | 118.9 | **361.84** | 63.8 | **501.69** | 218.2 |
| M18349cds#1_s_at | protein tyrosine phosphatase, receptor type, C | **99.87** | 13.4 | **243.42** | 65.8 | **142.37** | 7.1 | **172.56** | 58.0 |
| M22670cds_at | alpha-2-macroglobulin | **69.81** | 9.6 | **167.55** | 111.1 | **39.64** | 0.0 | **180.84** | 167.0 |
| K02814_at | kininogen 1 | **109.56** | 8.1 | **259.68** | 18.9 | **131.66** | 11.9 | **133.34** | 33.9 |
| K02814_g_at |  | **56.32** | 4.9 | **494.30** | 106.9 | **93.77** | 20.1 | **168.46** | 119.8 |
| rc_AA874924_at | lymphocyte antigen 86 (predicted) | **264.75** | 27.1 | **544.84** | 175.1 | **685.86** | 165.9 | **523.33** | 143.0 |
| U76206_at | purinergic receptor P2Y, G-protein coupled, 14 | **154.19** | 15.1 | **305.46** | 106.6 | **327.88** | 17.9 | **242.03** | 95.4 |
| rc_AA964003_s_at | arrestin, beta 2 | **137.52** | 20.4 | **202.45** | 97.0 | **42.82** | 1.6 | **156.84** | 113.1 |
| U42719_at | complement component 4a | **491.82** | 107.7 | **683.54** | 155.6 | **593.58** | 26.0 | **1056.68** | 402.3 |
| D30040_at | thymoma viral proto-oncogene 1 | **565.18** | 92.3 | **598.04** | 41.4 | **281.10** | 67.2 | **452.12** | 148.9 |
| U44845_at | vitronectin | **476.07** | 88.7 | **227.52** | 52.1 | **243.54** | 28.6 | **342.09** | 118.5 |
| U49062_at | CD24 antigen | **560.41** | 139.3 | **248.45** | 78.7 | **1719.18** | 45.1 | **900.56** | 592.2 |
| U49062_g_at |  | **209.91** | 24.0 | **77.72** | 3.8 | **238.75** | 2.3 | **189.99** | 49.5 |
