# Supplementary material for: Novel role for SLPI in MOG-induced EAE revealed by spinal cord expression analysis
Source: J Neuroinflammation. 2008 May 26;5:20. doi: 10.1186/1742-2094-5-20 (PMC2438345; doi:10.1186/1742-2094-5-20)
Supplement: Additional file 3 — Overview of "EAE-response CNS genes". Presentation of the normalised signal intensities of the "EAE-response CNS genes". For all genes, the differences between any disease phase and healthy rats are statistically significant according to the defined selection criteria. [file 1742-2094-5-20-S3.doc]

| Affymetrix ID | Gene Title | Gene Symbol | Av healthy | Av acute | Av recovery | Av relapsing |
| --- | --- | --- | --- | --- | --- | --- |
| rc_AI045023_at | --- | --- | 128.0 | 55.3 | 106.2 | 99.0 |
| M16112_at | calcium/calmodulin-dependent protein kinase II, beta | Camk2b | 152.2 | 66.3 | 76.1 | 61.8 |
| rc_AI009493_at | Transcribed locus | --- | 512.0 | 242.2 | 288.0 | 268.7 |
| rc_AI045554_at | Transcribed locus | --- | 43.1 | 15.7 | 36.0 | 38.1 |
| rc_AI058940_at | --- | --- | 390.7 | 94.4 | 96.3 | 190.0 |
| J04488_at | prostaglandin D2 synthase | Ptgds | 6208.4 | 885.3 | 1408.6 | 4329.5 |
| rc_AI111644_at | Transcribed locus | --- | 103.3 | 26.5 | 44.0 | 54.9 |
| rc_AI045919_at | plakophilin 4 | Pkp4 | 202.3 | 71.5 | 113.0 | 143.0 |
| X06984cds_s_at | aldolase C | Aldoc | 3516.7 | 849.2 | 1184.4 | 2062.2 |
| rc_AA850640_at | brain and acute leukemia, cytoplasmic | Baalc | 464.6 | 147.0 | 219.8 | 249.0 |
| M25638_s_at | neurofilament, light polypeptide | Nefl | 3169.4 | 491.1 | 826.0 | 1052.8 |
| rc_AA998575_at | Transcribed locus | --- | 113.8 | 16.0 | 38.1 | 54.6 |
| rc_AI044668_at | cartilage acidic protein 1 | Crtac1 | 108.4 | 29.7 | 58.1 | 59.7 |
| M63656_s_at | aldolase C | Aldoc | 8192.0 | 1552.1 | 3213.7 | 4067.7 |
| rc_AI235528_at | strongly similar to NP_035560.1 synuclein, gamma | --- | 803.4 | 187.4 | 290.0 | 276.3 |
| rc_AI102073_at | mal, T-cell differentiation protein 2 | Mal2 | 183.5 | 46.5 | 102.5 | 82.7 |
| rc_AI101203_at | similar to Brain specific membrane-anchored protein precursor | RGD1305557 | 1152.1 | 288.0 | 584.1 | 474.4 |
| rc_AI180066_at | Transcribed locus | --- | 820.3 | 176.1 | 421.7 | 304.4 |
| rc_AI232700_at | Transcribed locus | --- | 266.9 | 73.5 | 172.4 | 103.3 |
| rc_AA819900_at | protocadherin alpha subfamily C, 2 | Pcdhac2 | 421.7 | 113.0 | 276.3 | 129.8 |
| rc_AI175348_at | Receptor accessory protein 1 | Reep1 | 922.9 | 190.0 | 749.6 | 393.4 |
| rc_AI229935_at | similar to WARP | RGD1563152 | 626.0 | 117.0 | 333.1 | 252.5 |
| rc_AI146177_at | Transcribed locus | --- | 541.2 | 71.0 | 230.7 | 194.0 |
| rc_AI101943_at | strongly similar to NP_796210.2 plasma membrane calcium ATPase 3 | --- | 564.2 | 83.3 | 294.1 | 195.4 |
| rc_AI228050_at | Transcribed locus | --- | 1584.7 | 233.9 | 948.8 | 617.4 |
| rc_AI104773_at | CDNA clone IMAGE:7302289 | --- | 337.8 | 85.0 | 240.5 | 151.2 |
| rc_AA818602_at | Ras-like without CAAX 2 | Rit2 | 754.8 | 130.7 | 404.5 | 270.6 |
| rc_AI101350_at | TSPY-like 4 | Tspyl4 | 2998.4 | 512.0 | 1629.3 | 1184.4 |
| rc_AI170757_at | protocadherin alpha subfamily C, 2 | Pcdhac2 | 162.0 | 38.9 | 93.1 | 57.7 |
| rc_AI180254_at | --- | --- | 374.8 | 113.8 | 257.8 | 187.4 |
| rc_AI145880_at | Synapsin II | Syn2 | 765.4 | 136.2 | 448.8 | 288.0 |
| rc_AI227991_at | visinin-like 1 | Vsnl1 | 2033.9 | 481.0 | 1176.3 | 719.1 |
| rc_AI102369_at | adaptor-related protein complex 3, beta 2 subunit | Ap3b2 | 308.7 | 85.6 | 179.8 | 128.0 |
| rc_AI111796_at | elongation of very long chain fatty acids (FEN1/Elo2, SUR4/Elo3, yeast)-like 4 | Elovl4 | 56.5 | 18.6 | 28.1 | 17.1 |
| rc_AA819292_at | Transcribed locus | --- | 72.0 | 25.3 | 29.0 | 19.7 |
| rc_AI176740_at | zinc finger, DHHC domain containing 2 | Zdhhc2 | 122.8 | 32.4 | 43.1 | 28.4 |
| rc_AA818417_at | leucine rich repeat and fibronectin type III domain containing 5 | Lrfn5 | 74.5 | 20.5 | 24.4 | 18.1 |
| rc_AA943229_s_at | leucine-rich repeat LGI family, member 1 | Lgi1 | 113.0 | 37.0 | 99.7 | 104.0 |
| rc_AA942959_at | protein phosphatase 1, regulatory (inhibitor) subunit 1B | Ppp1r1b | 294.1 | 86.2 | 138.1 | 210.8 |
| rc_AI229198_at | Transcribed locus | --- | 138.1 | 20.4 | 259.6 | 145.0 |
| rc_AI228167_at | EST AI316813 | RGD1310827 | 955.4 | 86.2 | 572.1 | 643.6 |
| rc_AI235942_at | Aquaporin 4 | Aqp4 | 3956.5 | 724.1 | 3373.4 | 2856.4 |
| rc_AI227644_at | Transcribed locus | --- | 541.2 | 167.7 | 604.7 | 326.3 |
| rc_AI072020_at | Synapsin II | Syn2 | 505.0 | 91.1 | 367.1 | 306.6 |
| rc_AI180306_at | phytanoyl-CoA hydroxylase interacting protein-like | Phyhipl | 1910.9 | 340.1 | 2033.9 | 1176.3 |
| rc_AI233394_at | DIX domain containing 1 | Dixdc1 | 388.0 | 71.5 | 367.1 | 207.9 |
| rc_AI136114_at | monocyte to macrophage differentiation-associated 2 | Mmd2 | 855.1 | 100.4 | 699.4 | 418.8 |
| rc_AI112459_at | solute carrier organic anion transporter family, member 1c1 | Slco1c1 | 955.4 | 117.8 | 1136.2 | 754.8 |
| rc_AI072720_at | similar to KIAA1189 protein | RGD1308367 | 2876.3 | 278.2 | 3019.3 | 1530.7 |
| rc_AI170376_at | Transcribed locus | --- | 256.0 | 66.3 | 219.8 | 138.1 |
| rc_AI072459_at | Transcribed locus | --- | 61.0 | 19.2 | 50.6 | 33.8 |
| AF021923_at | solute carrier family 24 (sodium/potassium/calcium exchanger), member 2 | Slc24a2 | 415.9 | 92.4 | 415.9 | 238.9 |
| X17682_s_at | microtubule-associated protein 2 | Mtap2 | 119.4 | 45.9 | 206.5 | 117.0 |
| rc_AI235060_at | --- | --- | 252.5 | 72.5 | 328.6 | 212.3 |
| rc_AI144767_s_at | tropomyosin 1, alpha | Tpm1 | 213.8 | 104.0 | 182.3 | 128.0 |
| rc_AI014145_at | hypothetical protein LOC303515 | LOC303515 | 91.8 | 34.8 | 61.0 | 58.1 |
| rc_AI227905_at | abhydrolase domain containing 3 | Abhd3 | 168.9 | 15.7 | 70.5 | 48.8 |
| M27925_at | synapsin II | Syn2 | 176.1 | 52.7 | 108.4 | 79.9 |
| rc_AI009235_at | --- | --- | 130.7 | 45.9 | 95.7 | 91.1 |
| AF044201_at | Fas apoptotic inhibitory molecule 2 | Faim2 | 989.1 | 382.7 | 652.6 | 588.1 |
| M83196_at | microtubule-associated protein 1 A | Mtap1a | 1184.4 | 436.5 | 729.1 | 608.9 |
| rc_AA924526_at | G protein-coupled receptor, family C, group 5, member B | Gprc5b | 843.4 | 266.9 | 617.4 | 617.4 |
| AB003400_at | D-amino acid oxidase 1 | Dao1 | 182.3 | 21.6 | 88.6 | 92.4 |
| rc_AA943892_at | angiotensinogen (serpin peptidase inhibitor, clade A, member 8) | Agt | 797.9 | 120.3 | 342.5 | 436.5 |
| rc_AA997651_at | phytanoyl-CoA hydroxylase interacting protein-like | Phyhipl | 942.3 | 352.1 | 765.4 | 719.1 |
| rc_AI172335_at | --- | --- | 79.3 | 16.3 | 92.4 | 48.2 |
| rc_AA943463_at | Similar to AP2 associated kinase 1 | RGD1563580 | 522.8 | 256.0 | 464.6 | 364.6 |
| U63740_at | fasciculation and elongation protein zeta 1 | Fez1 | 1243.3 | 296.1 | 1082.4 | 709.2 |
| M12112mRNA#3_s_at | angiotensinogen (serpin peptidase inhibitor, clade A, member 8) | Agt | 484.4 | 86.2 | 451.9 | 390.7 |
| rc_AA943700_at | Transcribed locus | --- | 424.6 | 44.3 | 284.0 | 222.9 |
| rc_AI044120_at | Transcribed locus | --- | 38.6 | 19.8 | 15.5 | 39.9 |
| rc_AI233875_at | Similar to hedgehog-interacting protein | RGD1564108 | 196.7 | 41.1 | 53.4 | 56.9 |
| rc_AA818197_at | similar to protein Tyr phosphatase | LOC498331 | 167.7 | 102.5 | 61.8 | 75.1 |
| rc_AI059602_at | microtubule-associated protein 1b | Map1b | 56.1 | 24.8 | 13.3 | 27.1 |
| rc_AI228132_i_at | cerebral endothelial cell adhesion molecule 1 | Ceecam1 | 107.6 | 47.8 | 22.2 | 30.9 |
| rc_AI176699_at | Transcribed locus | --- | 266.9 | 67.2 | 57.3 | 69.6 |
| rc_AI229016_s_at | farnesyl diphosphate farnesyl transferase 1 | Fdft1 | 1067.5 | 418.8 | 421.7 | 467.9 |
| rc_AI235129_at | trafficking protein, kinesin binding 2 | Trak2 | 292.0 | 74.5 | 35.0 | 93.7 |
| rc_AI231782_at | --- | --- | 152.2 | 77.7 | 58.1 | 72.0 |
| H31285_at | Transcribed locus | --- | 413.0 | 116.2 | 64.4 | 149.1 |
| H32813_at | similar to hypothetical protein | RGD1305045 | 237.2 | 128.0 | 85.6 | 142.0 |
| rc_AI071867_at | phosphatidylserine synthase 2 | Ptdss2 | 274.4 | 121.9 | 95.0 | 173.6 |
| rc_AA819133_at | --- | --- | 855.1 | 270.6 | 250.7 | 415.9 |
| rc_AI229137_at | similar to RIKEN cDNA 6030419C18 gene | RGD1562618 | 115.4 | 40.2 | 35.0 | 41.4 |
| rc_AA818494_at | --- | --- | 1201.0 | 315.2 | 190.0 | 270.6 |
| rc_AI177155_at | NAD(P) dependent steroid dehydrogenase-like | Nsdhl | 439.6 | 192.7 | 168.9 | 110.7 |
| rc_AI233818_at | A kinase (PRKA) anchor protein (gravin) 12 | Akap12 | 942.3 | 556.4 | 530.1 | 461.4 |
| rc_AA997869_s_at | peptidyl arginine deiminase, type II | Padi2 | 57.3 | 24.9 | 13.3 | 35.0 |
| rc_AI029180_at | cell cycle progression 1 | Ccpg1 | 53.8 | 57.3 | 26.0 | 45.3 |
| X15705cds_at | heat shock 70kDa protein 2 | Hspa2 | 127.1 | 128.9 | 51.3 | 71.0 |
| rc_AI058941_s_at | dimethylarginine dimethylaminohydrolase 1 | Ddah1 | 142.0 | 179.8 | 55.3 | 73.5 |
| rc_AI060295_at | RAB5B, member RAS oncogene family | Rab5b | 68.6 | 64.4 | 29.2 | 40.5 |
| U23146cds_s_at | A kinase (PRKA) anchor protein (gravin) 12 | Akap12 | 142.0 | 128.9 | 47.2 | 65.8 |
| rc_AI012213_at | Protein phosphatase 2A, regulatory subunit B (PR 53) | Ppp2r4 | 261.4 | 268.7 | 106.2 | 187.4 |
| rc_AA899258_at | COP9 (constitutive photomorphogenic) homolog, subunit 7a | Cops7a | 160.9 | 167.7 | 38.6 | 101.1 |
| rc_AA998352_s_at | ras homolog gene family, member B | Rhob | 310.8 | 278.2 | 72.5 | 221.3 |
| rc_AA925126_at | transmembrane protein 55A | Tmem55a | 183.5 | 141.0 | 52.3 | 93.1 |
| rc_AI013149_at | melanoma antigen, family D, 2 | Maged2 | 81.6 | 72.0 | 20.7 | 44.6 |
| rc_AI013730_at | --- | --- | 247.3 | 222.9 | 86.8 | 146.0 |
| rc_AI031038_at | similar to RIKEN cDNA 1110067D22 (predicted) | RGD1307414_predicted | 62.2 | 60.1 | 17.6 | 39.9 |
| X74293_s_at | integrin alpha 7 | Itga7 | 60.1 | 44.0 | 18.5 | 27.1 |
| AF086758_g_at | solute carrier family 12, member 2 | Slc12a2 | 94.4 | 68.6 | 32.2 | 42.5 |
| rc_AA957486_at | Transcribed locus | --- | 111.4 | 87.4 | 54.9 | 68.1 |
| M15883_at | clathrin, light polypeptide (Lcb) | Cltb | 213.8 | 150.1 | 93.7 | 127.1 |
| rc_AI639532_at | troponin C type 2 (fast) | Tnnc2 | 95.0 | 22.2 | 18.5 | 38.6 |
| AB016161UTR#1_g_at | gamma-aminobutyric acid (GABA) B receptor 1 | Gabbr1 | 398.9 | 194.0 | 166.6 | 254.2 |
| U49953_s_at | p21 (CDKN1A)-activated kinase 1 | Pak1 | 177.3 | 81.0 | 65.8 | 80.4 |
| M76426_at | dipeptidylpeptidase 6 | Dpp6 | 256.0 | 138.1 | 95.0 | 128.0 |
| Z35654_at | mcf.2 transforming sequence-like | Mcf2l | 112.2 | 49.9 | 28.4 | 41.1 |
| rc_AI013466_at | similar to FKSG26 protein (predicted) | RGD1309054_predicted | 568.1 | 380.0 | 184.8 | 280.1 |
| AF009603_at | SH3-domain GRB2-like 2 | Sh3gl2 | 374.8 | 173.6 | 89.3 | 151.2 |
| AF091834_at | N-ethylmaleimide sensitive fusion protein | Nsf | 292.0 | 82.7 | 16.8 | 57.3 |
| M27726_at | brain glycogen phosphorylase | Pygb | 474.4 | 140.1 | 42.2 | 134.4 |
| rc_AA850890_at | imprinted and ancient | Impact | 213.8 | 117.8 | 64.0 | 95.0 |
| AF083330_at | kinesin family member 3C | Kif3c | 120.3 | 71.5 | 52.0 | 67.2 |
| rc_AA964146_s_at | similar to RIKEN cDNA 2610019F03 | LOC498662 | 199.5 | 96.3 | 68.6 | 109.9 |
| rc_AI029183_s_at | gap junction membrane channel protein alpha 1 | Gja1 | 149.1 | 66.7 | 19.6 | 61.0 |
| rc_AI059084_at | similar to RIKEN cDNA 2310022B05 | RGD1559896 | 224.4 | 154.3 | 101.1 | 171.3 |
| rc_AA964882_at | --- | --- | 120.3 | 71.0 | 42.2 | 72.0 |
| Z49858_at | plasma membrane proteolipid | Pllp | 843.4 | 390.7 | 347.3 | 505.0 |
| D90048exon_g_at | ATPase, Na+/K+ transporting, beta 2 polypeptide | Atp1b2 | 340.1 | 64.4 | 45.3 | 141.0 |
| rc_AI010499_at | --- | --- | 93.1 | 40.8 | 34.5 | 61.8 |
| M95591_at | farnesyl diphosphate farnesyl transferase 1 | Fdft1 | 548.7 | 247.3 | 100.4 | 162.0 |
| Y17048_at | calcium binding protein 1 | Cabp1 | 245.6 | 104.0 | 113.0 | 130.7 |
| rc_AA850692_at | Membrane associated guanylate kinase, WW and PDZ domain containing 2 | Magi2 | 138.1 | 75.1 | 57.3 | 74.0 |
| D10666_at | visinin-like 1 | Vsnl1 | 584.1 | 226.0 | 259.6 | 268.7 |
| rc_AA943576_at | Similar to CD2-associated protein (predicted) | RGD1305090 | 643.6 | 317.4 | 326.3 | 374.8 |
| rc_AA894317_s_at | chimerin (chimaerin) 1 | Chn1 | 349.7 | 156.5 | 133.4 | 151.2 |
| M95591_g_at | farnesyl diphosphate farnesyl transferase 1 | Fdft1 | 173.6 | 22.5 | 16.8 | 20.1 |
| U78977_g_at | similar to Potential phospholipid-transporting ATPase IIB | LOC291411 | 873.1 | 390.7 | 335.5 | 407.3 |
| S50879_at | acetylcholinesterase | Ache | 196.7 | 73.0 | 64.9 | 79.3 |
| D70817_at | complexin 1 | Cplx1 | 922.9 | 302.3 | 272.5 | 362.0 |
| X06889cds_at | RAB3A, member RAS oncogene family | Rab3a | 776.0 | 195.4 | 186.1 | 317.4 |
| M21410_s_at | 5-hydroxytryptamine (serotonin) receptor 2C | Htr2c | 58.5 | 41.9 | 27.3 | 25.8 |
| rc_AI045232_at | PFTAIRE protein kinase 1 | Pftk1 | 40.2 | 25.1 | 13.3 | 17.9 |
| rc_AI070489_at | Transcribed locus | --- | 261.4 | 126.2 | 704.3 | 218.3 |
| AA848702_at | Platelet-derived growth factor, D | Pdgfd | 118.6 | 64.4 | 250.7 | 115.4 |
| rc_AI101014_at | Glutaredoxin 2 (thioltransferase) | Glrx2 | 113.8 | 67.6 | 259.6 | 136.2 |
| rc_AI237207_at | Inhibitor of DNA binding 4 | Id4 | 143.0 | 31.8 | 461.4 | 132.5 |
| rc_AA892128_at | peroxisomal biogenesis factor 11A | Pex11a | 142.0 | 136.2 | 286.0 | 195.4 |
| rc_AA964481_at | Plakophilin 4 | Pkp4 | 51.6 | 61.0 | 114.6 | 75.1 |
| rc_AA957117_at | Cadherin 22 | Cdh22 | 172.4 | 188.7 | 515.6 | 249.0 |
| rc_AA892296_at | homeo box B8 (mapped) | Hoxb8_mapped | 171.3 | 138.1 | 367.1 | 221.3 |
| X57764_s_at | endothelin receptor type B | Ednrb | 232.3 | 125.4 | 792.4 | 326.3 |
| S65355_at | endothelin receptor type B | Ednrb | 158.7 | 99.0 | 617.4 | 252.5 |
| rc_AI013671_at | Transcribed locus | --- | 30.9 | 113.8 | 129.8 | 58.9 |
| rc_AA866465_s_at | Transcribed locus | --- | 27.9 | 54.2 | 118.6 | 37.3 |
| rc_AA945867_at | Jun oncogene | Jun | 97.0 | 162.0 | 294.1 | 153.3 |
| rc_AA859977_at | similar to Heat shock protein HSP 90-beta | HSP84 | 83.9 | 110.7 | 296.1 | 121.1 |
| rc_AA944014_at | Jun oncogene | Jun | 58.1 | 111.4 | 288.0 | 82.7 |
| M58369_at | pancreatic lipase | Pnlip | 226.0 | 179.8 | 1152.1 | 867.1 |
| rc_AA958001_at | collagen triple helix repeat containing 1 | Cthrc1 | 26.4 | 50.2 | 77.2 | 52.7 |
| rc_AI639108_at | --- | --- | 77.2 | 107.6 | 188.7 | 144.0 |
| rc_AA926213_g_at | gamma-aminobutyric acid (GABA) B receptor 1 | Gabbr1 | 227.5 | 280.1 | 508.5 | 530.1 |
| rc_AA945932_at | Annexin3 | --- | 107.6 | 182.3 | 388.0 | 317.4 |
| rc_AA963077_at | Transcribed locus | --- | 36.8 | 64.9 | 51.6 | 75.6 |
| M22670cds_at | alpha-2-macroglobulin | A2m | 45.9 | 113.8 | 18.1 | 57.7 |
| rc_AI012418_at | Sterol O-acyltransferase 1 | Soat1 | 28.4 | 83.3 | 28.2 | 45.3 |
| U59801_at | integrin alpha M | Itgam | 61.0 | 237.2 | 41.9 | 76.1 |
| Z54212_at | epithelial membrane protein 1 | Emp1 | 101.8 | 288.0 | 60.5 | 109.1 |
| U82612cds_g_at | fibronectin 1 | Fn1 | 74.5 | 996.0 | 19.2 | 54.2 |
| rc_AI169311_at | similar to hypothetical protein MGC34760 | RGD1359349 | 29.4 | 194.0 | 86.2 | 56.1 |
| rc_AI230625_at | TC-1/C8orf4 | --- | 17.9 | 72.0 | 43.4 | 27.9 |
| rc_AI012235_at | chemokine (C-X-C motif) ligand 11 | Cxcl11 | 16.1 | 639.1 | 183.5 | 28.8 |
| rc_AI233219_at | endothelial cell-specific molecule 1 | Esm1 | 22.6 | 116.2 | 16.9 | 19.6 |
| K02814_g_at | kininogen 1 | Kng1 | 44.0 | 306.6 | 68.6 | 97.0 |
| S66184_s_at | lysyl oxidase | Lox | 46.5 | 261.4 | 63.1 | 60.1 |
| rc_AA964867_at | Guanine deaminase | Gda | 42.2 | 121.9 | 44.3 | 45.9 |
| rc_AI071531_s_at | oxidized low density lipoprotein (lectin-like) receptor 1 | Oldlr1 | 99.0 | 367.1 | 164.3 | 138.1 |
| M25073_at | alanyl (membrane) aminopeptidase | Anpep | 72.5 | 474.4 | 135.3 | 144.0 |
| X57018_at | Gardner-Rasheed feline sarcoma viral (Fgr) oncogene homolog | Fgr | 56.1 | 328.6 | 91.1 | 77.2 |
| rc_AA946503_at | lipocalin 2 | Lcn2 | 21.7 | 498.0 | 70.5 | 48.2 |
| V01216_at | orosomucoid 1 | Orm1 | 33.8 | 131.6 | 48.2 | 38.1 |
| rc_AA945996_at | angiotensin receptor-like 1 | Agtrl1 | 75.6 | 471.1 | 196.7 | 300.2 |
| rc_AI072476_at | Matrix metalloproteinase 19 | Mmp19 | 20.3 | 104.7 | 109.9 | 80.4 |
| rc_AI232295_at | glycoprotein 49b | Gp49b | 32.0 | 1379.6 | 393.4 | 190.0 |
| rc_AI010468_at | Similar to CD300A/igsf12 | --- | 210.8 | 430.5 | 349.7 | 335.5 |
| rc_AI013502_at | Secretory carrier membrane protein 1 | Scamp1 | 114.6 | 380.0 | 224.4 | 302.3 |
| U56241_at | v-maf musculoaponeurotic fibrosarcoma oncogene family, protein B | Mafb | 17.5 | 71.5 | 41.4 | 50.9 |
| AB000778_s_at | phospholipase D1 | Pld1 | 86.8 | 352.1 | 164.3 | 162.0 |
| rc_AI045955_at | similar to Clecsf12/Dectin1 | --- | 19.7 | 1152.1 | 280.1 | 238.9 |
| rc_AI059916_f_at | signal transducer and activator of transcription 3 | Stat3 | 35.5 | 101.1 | 91.8 | 86.2 |
| rc_AA945278_f_at | Complement component factor h-like 1 | Cfhl1 | 121.1 | 498.0 | 461.4 | 512.0 |
| S56464mRNA_g_at | Hexokinase II | HkII | 39.1 | 404.5 | 584.1 | 288.0 |
